# Learning-enabled collaborative intelligence in search and rescue robotics: a survey of paradigms, models, and challenges

**DOI:** 10.3389/frobt.2026.1832982

**Published:** 2026-10-14

**Authors:** M. I. R. Shuvo, Awal Ahmed Fime, Raiyan Ashraf, Nafiul Alam, Jong-Hoon Kim

**Affiliations:** Advanced Telerobotic Research Lab, Computer Science, Kent State University, Kent, OH, United States

**Keywords:** explainable AI, federated learning, human-robot collaboration, knowledge transfer, large language models, multi-agent learning, search and rescue robotics

## Abstract

Search and Rescue (SAR) robotics increasingly depends on collaborative intelligence to operate effectively in dynamic, uncertain, and time-sensitive environments. Modern SAR missions require coordinated interaction among heterogeneous robotic agents as well as structured collaboration between humans and intelligent systems for perception, decision-making, and adaptive mission execution. This paper presents a structured narrative review supported by a systematic literature search of learning-enabled collaborative intelligence frameworks in SAR robotics, organized around three paradigms: human-to-machine (H2M), machine-to-machine (M2M), and machine-to-human (M2H) learning. The review examines recent developments in reinforcement learning, federated and decentralized learning, knowledge distillation, swarm intelligence, semantic communication, and the growing integration of large language models (LLMs) and vision-language models (VLMs) for knowledge grounding, transfer, and explainability. Beyond algorithmic advances, the analysis emphasizes deployment-oriented challenges including partial observability, communication constraints, uncertainty management, model reliability, and real-time operational requirements. Rather than conducting a fully PRISMA-compliant systematic review, this work synthesizes representative and influential studies to identify emerging trends, architectural trade-offs, deployment limitations, and future research directions for scalable and trustworthy collaborative SAR systems.

## 1 Introduction

### 1.1 Background of search and rescue (SAR) robotics

Disasters, whether natural or caused by humans, incur significant societal, economic, and environmental costs. Since 1990, disasters have impacted over 4.4 billion individuals, resulted in about 1.3 million fatalities, and incurred losses above $2.97 trillion for Disaster Risk Reduction (2023). Natural disasters like earthquakes and floods, together with industrial mishaps, compromise public health through injuries, post-traumatic stress, and illness, while simultaneously harming ecosystems through pollution and deforestation. Displacement, loss of livelihood, and heightened inequality especially among at-risk populations–are prevalent outcomes (Morrissey and Reser, 2007). As climate change and urbanization intensify threats, the need for effective and adaptive disaster response strategies becomes paramount.

Robotic-assisted search and rescue (SAR) improves disaster response by coordinating ground-based, aerial, and marine robots. Ground robots function in hazardous environments for debris removal and logistic assistance, aerial drones facilitate damage assessment, survivor identification, and hazard mapping, while aquatic robots assist in aquatic rescue operations (Murphy and Burke, 2005). Progress in machine learning (ML), deep learning (DL), and large language models (LLMs) facilitates autonomous perception, navigation, and coordination, thus reducing human danger and enhancing situational awareness (Apvrille et al., 2014). Supervised, unsupervised, reinforcement, federated, transfer, and self-supervised learning provide adaptation with less supervision, but large language models enhance mission interpretation, multimodal reasoning, and adaptive decision-making. However, achieving robust, reliable, and real-time autonomy in highly dynamic SAR environments remains challenging, motivating hybrid AI frameworks that integrate sensor fusion, federated learning, uncertainty-aware modeling, and explainable AI.

### 1.2 Motivation for the study

When there is a disaster, heterogeneous robots equipped with sensors such as cameras, microphones, LiDAR, and thermal sensors can investigate hazardous areas to support SAR operations. Human operators remain responsible for mission supervision and intervene when needed. For SAR to work effectively, robots need to learn from humans (H2M), learn, share, and validate knowledge with other robots (M2M), and communicate acquired knowledge back to human operators (M2H). Because disaster areas change quickly and unpredictably, robots must continuously acquire new information, adapt their behavior, and make reliable decisions. A key challenge is enabling continuous learning while maintaining accuracy, robustness, and flexibility in high-risk scenarios.

Traditional human-to-machine (H2M) learning approaches, including supervised learning, imitation learning, and deep reinforcement learning, frequently encounter difficulties in SAR because of their dependence on structured input and static training, which restricts generalization in cluttered, smoke-filled, or partially observable situations. Machine-to-machine (M2M) learning addresses part of this issue by enabling the exchange and cross-validation of semantic knowledge among robots, thereby reducing human workload and accelerating response. However, it also introduces risks related to uncertainty, semantic drift, and the propagation of erroneous information, requiring robust validation mechanisms. Effective M2M learning further requires machine-to-human (M2H) communication, which provides interpretable and confidence-aware updates to support human decision-making. This review examines H2M, M2M, and M2H learning in robotic SAR systems, focusing on techniques, deployment challenges, and prospective developments, as depicted in Figure 1. The review is guided by questions such as: “What are the current H2M, M2M, and M2H methods?“, “How do they address real-world challenges?“, and “What gaps remain in ensuring effective learning between humans and autonomous systems?”

**FIGURE 1:**
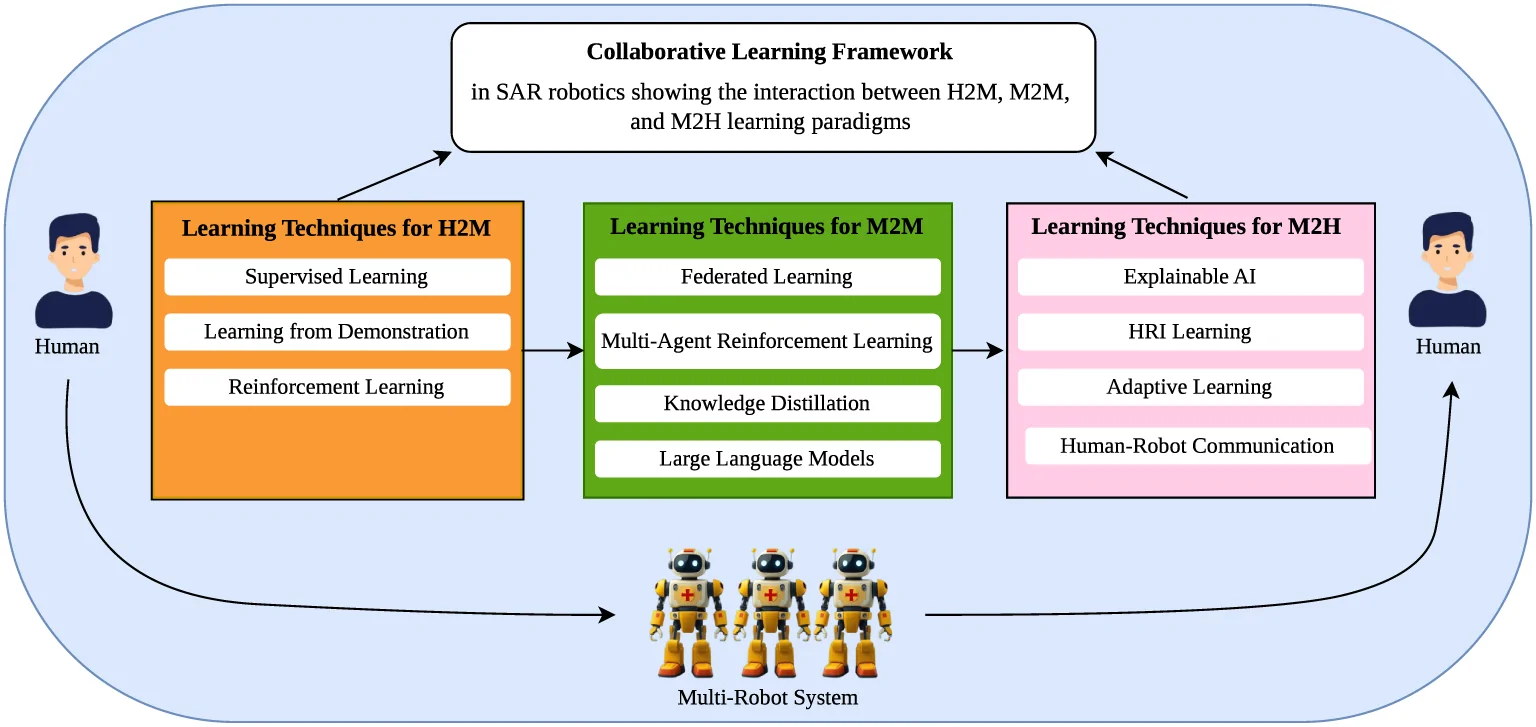
Conceptual survey framework of the proposed research, illustrating the H2M/M2M/M2H collaborative learning paradigms and the associated learning techniques and interactions within a multi-robot SAR system.

These questions are not addressed in isolation but through the lens of cross-paradigm interaction, examining not only what each learning paradigm contributes individually, but also how failures and constraints in one paradigm propagate into the others. Unlike prior surveys that treat human-to-machine (H2M), machine-to-machine (M2M), and machine-to-human (M2H) learning as largely separate concerns, this review considers them as interdependent layers of a collaborative SAR intelligence pipeline. The quality of H2M training directly constrains the knowledge available for M2M exchange, while the effectiveness of M2H communication determines whether human operators can appropriately supervise and validate machine-generated knowledge. This perspective reveals architectural gaps that are difficult to identify through single-paradigm analyses, including the absence of end-to-end uncertainty propagation across learning layers and the lack of standardized interfaces between symbolic M2M representations and human-interpretable M2H outputs. Consequently, the H2M/M2M/M2H framework serves not only as an organizational structure but also as an analytical lens for examining inter dependencies, failure modes, and open research challenges in collaborative SAR intelligence.

This paper is positioned as a structured narrative review supported by a systematic literature search rather than a fully PRISMA-compliant systematic review. The objective is to synthesize representative and influential studies across H2M, M2M, and M2H learning paradigms, with emphasis on conceptual organization, deployment trade-offs, and research gaps in collaborative SAR intelligence. Therefore, the review focuses on cross-paradigm interpretation and practical implications rather than quantitative meta-analysis or exhaustive statistical comparison.

Despite significant advances in machine learning for robotics, collaborative knowledge transfer among heterogeneous robots in search and rescue scenarios remains challenging. Existing approaches often assume stable communication infrastructure, homogeneous sensing capabilities, and consistent environmental conditions. However, real-world disaster environments introduce intermittent connectivity, non-IID data distributions, and heterogeneous robotic platforms. These limitations highlight the need for robust frameworks that enable reliable knowledge transfer and collaborative learning across distributed robotic agents.

The key motivations of this study are as follows.

- Enabling collaborative and symbolic learning in SAR
- Enabling robots to acquire task-relevant knowledge from humans through supervised, imitation, reinforcement, and multimodal learning in uncertain SAR environments.
- Supporting M2M learning via semantic prompt exchange, geo-symbolic representations, and federated learning to enable decentralized adaptation and situational awareness.
- Facilitating interpretable M2H communication through explainable LLM-based summaries of mission status, object understanding, and environmental changes.
- Ensuring reliable and interpretable knowledge propagation
- Improving reasoning under degraded perception using symbolic object descriptors and geometry-aware relational modeling.
- Validating transferred knowledge through confidence estimation, cross-agent semantic agreement, and prompt-grounded feedback to prevent unstable or hallucinated inferences.
- Promoting trust and accountability via verifiable LLM outputs and explainable decision traces aligned with human-understandable representations.
- Reducing operational risk through continual adaptation
- Supporting continual, context-aware refinement of models and symbolic world representations while preserving critical prior knowledge.
- Enhancing robustness by aggregating diverse cross-agent observations and using domain-specific prompts for improved generalization.
- Strengthening coordination and trust through proactive explanation and human-aligned summarization of newly acquired knowledge.

The review framework is as follows: Section 2 describes the literature selection strategy, including paper collection, screening, and information extraction. Section 3 addresses recent developments in human-to-machine learning for SAR. Section 4 discusses machine-to-machine learning, emphasizing knowledge transfer, reliability, and deployment constraints. Section 5 analyzes machine-to-human learning methodologies. Section 6 discusses open issues and future research directions. Section 7 presents the limitations of this review, and Section 8 concludes the paper. Figure 2 presents the research roadmap for building intelligent robotic systems capable of SAR operations.

**FIGURE 2:**
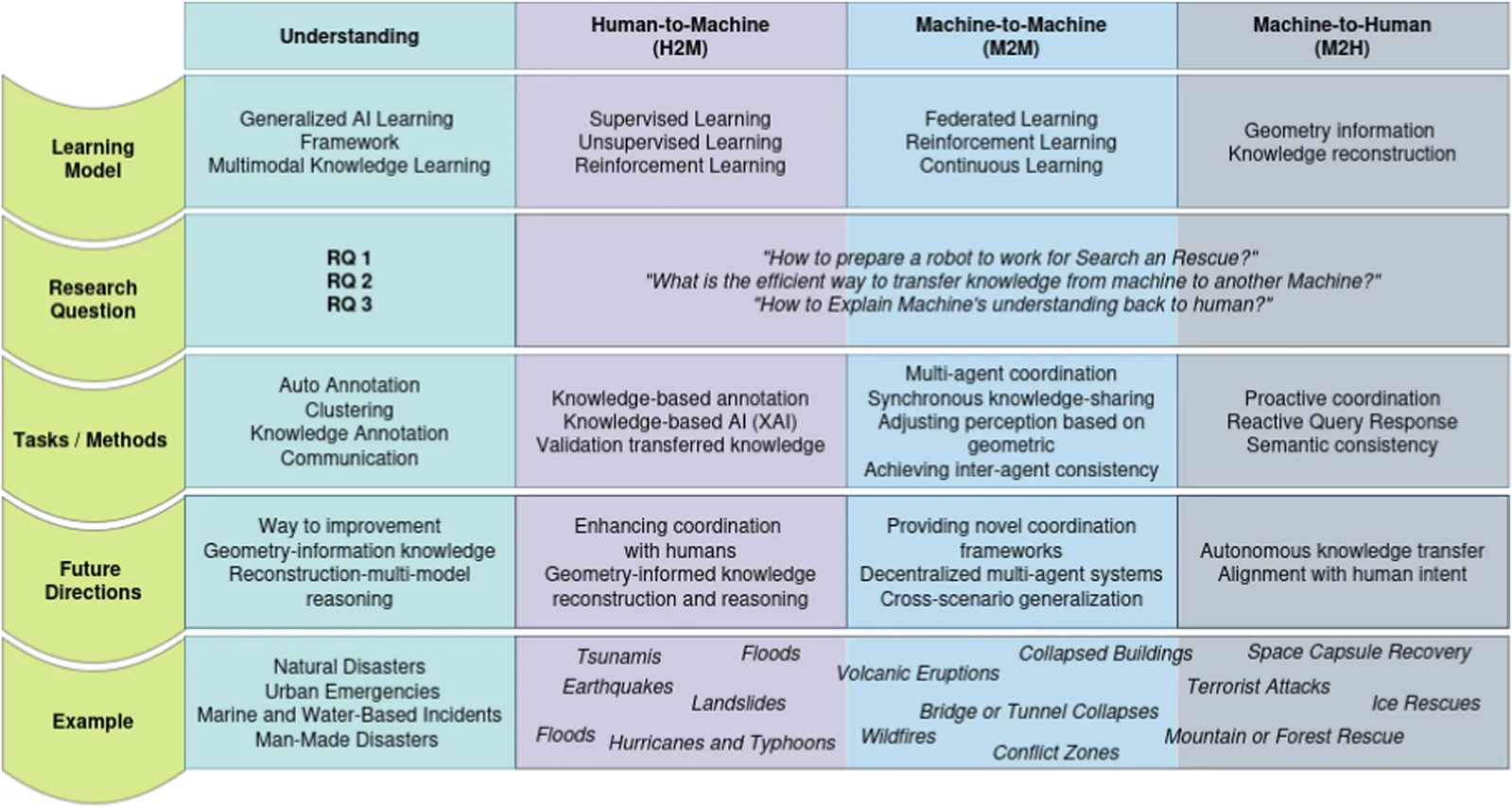
Roadmap of the literature analysis focused on the three examined collaborative learning paradigms, namely, human-to-machine, machine-to-machine, and machine-to-human learning in search and rescue robotics.

## 2 Literature selection strategy

### 2.1 Paper collection

To improve transparency and reproducibility, this review employed a structured literature search and screening strategy to identify representative studies related to collaborative intelligence in SAR robotics. Although the review is not intended as a fully PRISMA-compliant systematic review, systematic search and screening procedures were applied to improve methodological consistency and reduce selection bias.

To collect relevant literature for this review, we conducted a comprehensive search across multiple academic databases, including IEEE Xplore, ACM Digital Library, Scopus, SpringerLink, Google Scholar, PubMed, NCBI, CINAHL, and Web of Science, using a variety of keywords such as “Search and Rescue Robot”, “Human-to-robot communication”, “Robot-to-robot communication”, “Machine-to-Machine Learning”, “Recent Techniques to Teach Robot”, “Interface for robot communication”, and “SAR dataset for robot.” Boolean combinations of SAR-related, collaborative-learning, and robotic-intelligence keywords were used to improve search relevance and coverage. Representative Boolean search strings included: (“search and rescue” AND “robot” AND “federated learning”), (“SAR robotics” AND “multi-agent reinforcement learning”), (“human-robot interaction” AND “explainable AI” AND “search and rescue”), (“large language model” AND “robot” AND “collaborative intelligence”), and (“machine-to-machine learning” AND “autonomous systems”). These strings were adapted across databases to conform to platform-specific syntax requirements. This survey reviews literature published between 2005 and 2025, covering foundational SAR robotics research as well as recent advances in multi-agent learning, federated systems, and large language model-based collaborative intelligence. While earlier works establish the foundations of SAR robotics and human-robot interaction, the review places particular emphasis on post-2019 developments and post-2022 LLM/VLM-based approaches.

Only English-language peer-reviewed journal and conference papers directly related to collaborative intelligence, SAR robotics, multi-agent learning, explainable AI, or human-robot interaction were considered for inclusion. Non-SAR-focused studies, duplicate publications, purely hardware-oriented works without collaborative intelligence components, editorials, and papers lacking sufficient methodological detail were excluded from the final synthesis.

Initially, we selected papers based on their titles to assess relevance. If the title was unclear, we reviewed the abstract to determine whether the article warranted further examination. Following title and abstract screening, full-text assessment was conducted to evaluate relevance to H2M, M2M, and M2H collaborative learning paradigms. The final paper selection emphasized representative, influential, and conceptually diverse studies related to collaborative SAR intelligence. Then we followed the steps mentioned in Figure 3 to ensure that only the most relevant and high-quality studies were included in the final survey. It should be noted that a small number of works from the authors’ research group are included in the reviewed corpus where they represent direct and relevant contributions to the collaborative SAR learning paradigms examined; these are cited on the same basis as all other included studies and do not constitute a systematic bias in the selection process.

**FIGURE 3:**
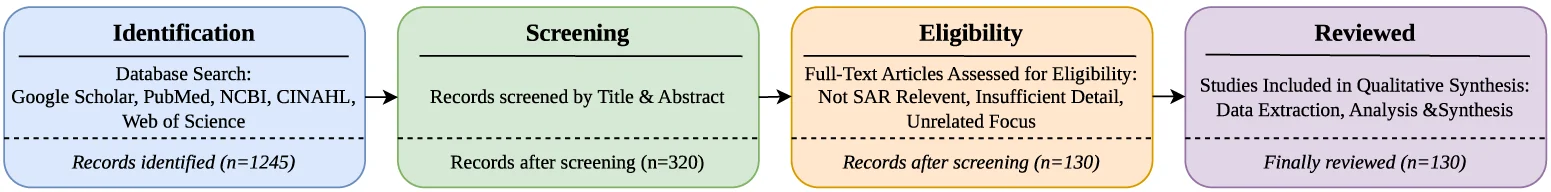
Workflow of the Literature Search, Screening, and Study Selection Process Used in this Structured Narrative Review.

Table 1 summarizes the overall literature search and screening strategy used in this review, including databases searched, keyword design, inclusion and exclusion criteria, screening stages, and study-selection considerations applied during the qualitative synthesis process.

**TABLE 1:** Literature search and screening strategy used in this review.

Category	Description
Databases searched	IEEE xplore, ACM digital library, scopus, SpringerLink, google scholar, PubMed, NCBI, CINAHL, and web of science
Publication year range	2005–2025, with emphasis on post-2019 collaborative learning approaches and post-2022 LLM/vlm-based methods
Language restriction	English-language publications only
Example search keywords	“Search and rescue robot”, “human-to-robot communication”, “robot-to-robot communication”, “machine-to-machine learning”, “human-robot interaction”, “federated learning SAR”, “LLM robotics”, “VLM robotics”, “semantic communication”, and “SAR dataset for robot”
Search logic	Boolean combinations using AND/OR operators across SAR-related, collaborative-learning, and robotic-intelligence keywords to improve search relevance and coverage; representative strings included: (“search and rescue” AND “robot” AND “federated learning”), (“SAR robotics” AND “multi-agent reinforcement learning”), and (“large language model” AND “robot” AND “collaborative intelligence”)
Inclusion criteria	Peer-reviewed journal and conference papers related to SAR robotics, collaborative intelligence, multi-agent learning, explainable AI, human-robot interaction, and semantic communication
Exclusion criteria	Duplicate studies, non-English papers, non-SAR-focused studies, purely hardware-oriented works without collaborative intelligence components, editorials, and papers lacking sufficient methodological detail
Duplicate removal	Potential duplicate entries encountered across multiple databases were checked manually during the screening process. As this study followed a structured narrative review methodology rather than a formal systematic-review protocol, duplicate counts were not recorded separately
Screening stages	Title screening, abstract screening, followed by full-text relevance assessment
Selection strategy	Final paper selection emphasized representative, influential, and conceptually diverse studies across H2M, M2M, and M2H learning paradigms
Review objective	Structured narrative review supported by systematic literature search and qualitative synthesis rather than quantitative meta-analysis

Figure 3 presents the workflow used to organize the literature search, screening, and qualitative study selection in this structured narrative review. The figure summarizes the methodology described in Table 1 and is intended to provide transparency regarding the review procedure rather than quantitative accounting of records or full PRISMA-compliant reporting.

### 2.2 Information extraction

We carefully reviewed the selected manuscripts focusing on key information including datasets, data preparation strategies, feature extraction methods, model architectures, evaluation protocols, reported performance, strengths, weaknesses, and deployment assumptions. To support structured qualitative synthesis, the selected studies were further analyzed based on their relevance to H2M, M2M, and M2H collaborative learning paradigms, communication strategies, explainability mechanisms, uncertainty handling, multi-agent coordination approaches, and real-world SAR deployment considerations. For the reviewed studies, we extracted information including author details, publication year, sensing scenario, datasets or simulation platforms, number of samples, sensing modalities, communication methods, evaluation metrics, robustness considerations, and the role of human interaction within the collaborative learning framework. This information was used to compare representative approaches, identify recurring limitations, and analyze deployment-oriented trade-offs across collaborative SAR intelligence systems.

## 3 Learning process for human-to-machine learning

Human-to-Machine (H2M) learning in SAR encompasses supervised learning, learning from demonstration, reinforcement learning, and LLMs, enabling robots to acquire perception, navigation, and decision-making capabilities under human guidance. These paradigms allow robots to adapt to complex disaster environments using labeled data, expert demonstrations, interaction-driven optimization, and high-level semantic reasoning.

### 3.1 Supervised learning

Supervised learning is extensively employed in SAR for perception tasks including survivor detection, object recognition, and structural damage evaluation. Object detection frameworks utilizing YOLO, Faster R-CNN, and SSD have been assessed across aerial, terrestrial, and thermal imagery, revealing trade-offs among accuracy, inference speed, and robustness in cluttered environments (Sambolek and Ivasic-Kos, 2021; Dong et al., 2021; Jadeja et al., 2024; Valarmathi et al., 2023). Recent research has concentrated on large scale-adaptive architectures and domain-specific improvements to enhance detection reliability in Synthetic Aperture Radar applications, encompassing unmanned aerial vehicle (UAV)-based maritime search (Xu J. et al., 2024), drone-assisted localization devoid of infrastructure (Albanese et al., 2021), and multi-task detection-segmentation models for assessing damaged structures (Surojaya et al., 2024). In addition to vision, supervised deep learning has been utilized for ground-penetrating radar reconstruction with GAN-augmented data, facilitating enhanced subsurface comprehension of collapsed structures (Hu et al., 2022). Swarm-based SAR systems amalgamate supervised perception with tracking and coordination techniques to augment multi-drone resilience in GPS-denied situations (Sangeeta et al., 2023; Redmon et al., 2016; Yeom, 2024).

Across these studies, a recurring finding is that detection accuracy degrades more sharply under thermal noise, smoke, and structural occlusion than under simple lighting variation, suggesting that domain-specific training data and modality selection are more consequential for SAR deployment reliability than architectural choices alone.

### 3.2 Learning from demonstration

Learning from Demonstration (LfD), also known as imitation learning, lets SAR robots learn complicated actions by copying expert demonstrations. This cuts down on manual programming and speeds up deployment. LfD has been utilized in collaborative manipulation, multi-UAV coordination, navigation, and communication during disaster scenarios (Wang et al., 2021; Wan et al., 2023; Shamsoshoara et al., 2021). Techniques like behavioral cloning, DAgger, and Gaussian mixture models let robots learn how to avoid obstacles, plan paths, and do tasks by copying what people do, but how well they do this relies on how well the demonstrations are done and how well the sensors cover the area (Cèsar-Tondreau et al., 2021; Tsai et al., 2021). Human skill makes high-stress SAR scenarios vulnerable to operator tiredness or cognitive overload, which can lead to unsatisfactory demonstrations. Recent frameworks filter planned rescue efforts from random human errors. Inverse reinforcement learning (IRL) and confidence-based filtering help robots learn rescue maneuver intent rather than motor actions, which may be faulty. Imitation learning with LiDAR and pedestrian-aware confidence maps has made navigation safer and more socially acceptable by making it more robust in changing environments (Qin et al., 2021; Yan et al., 2022). Recent advancements have expanded imitation learning to encompass perception and collaboration, incorporating small-object detection, object-centric policy learning, and human-robot assembly coordination (Yuan et al., 2023; Zhu et al., 2023; Zhang et al., 2022).

Taken together, these studies suggest that the practical ceiling of LfD in SAR is set less by algorithmic sophistication than by the quality and safety of the demonstration environment itself–a constraint that becomes particularly difficult to address when expert operators are unavailable, fatigued, or working under the same hazardous conditions the robot is meant to navigate.

### 3.3 Reinforcement learning

Through contact with dynamic and uncertain surroundings, reinforcement learning (RL) allows SAR robots to adapt, facilitating task allocation, exploration, and navigation. Satellite imagery, GNSS-denied multi-drone coordination, and goal-driven aerial localization have all been used to optimize coverage using RL-based frameworks, which have shown greater efficiency and adaptability than heuristic methods Gruffeille et al. (2024); Hickling et al. (2025); Pirinen et al. (2022)). In SAR deployments, human-centered RL designs enhance operator trust and usefulness even more (Ramezani et al., 2024). Recent methods use autoencoders and frontier-based exploration to decouple perception from policy learning, improving training efficiency while preserving performance (Ewers et al., 2025b; Niroui et al., 2019). Through adaptive speed control, cooperative learning, and disturbance-resistant controllers, RL has also been used in vehicle control and mission planning, enhancing robustness in UAV and unmanned surface vehicle (USV) SAR operations (Wang Y. et al., 2023; Wu et al., 2023).

What these results collectively indicate is that RL is most reliable in SAR when the operational environment during deployment resembles the simulation environment during training–a condition that is rarely guaranteed in real disaster scenarios, and that points to reward shaping and sim-to-real transfer as the field’s most pressing unsolved problems rather than policy architecture.

### 3.4 Comparative insights for SAR deployment

Human-to-machine (H2M) learning paradigms exhibit distinct strengths and limitations when deployed in real-world search and rescue (SAR) operations. Disaster environments are frequently characterized by partial observability caused by debris, smoke, unstable structures, and sensor noise. These conditions affect both the reliability of perception models and the robustness of learned control policies, making the choice of learning paradigm highly dependent on data availability, adaptability requirements, and real-time constraints. Table 2 summarizes key trade-offs across supervised learning, learning from demonstration (LfD), and reinforcement learning (RL) under typical SAR deployment conditions.

**TABLE 2:** Comparison of human-to-machine learning paradigms for search and rescue robotics.

Learning paradigm	Primary strength	Key limitation	Deployment readiness	SAR environmental challenges	Typical SAR applications
Supervised learning	High perception accuracy when sufficient labeled data are available	Requires large annotated datasets and may struggle with noisy or incomplete data	High	Occlusion, smoke, dust, limited labeled disaster data	Victim detection, terrain classification, hazard recognition
Learning from demonstration (LfD)	Enables intuitive policy learning from expert behavior	Limited availability of expert demonstrations and sim-to-real transfer challenges	Medium	Difficulty collecting demonstrations in hazardous environments	Navigation in cluttered environments, manipulation, human-guided task execution
Reinforcement learning (RL)	Adaptive decision-making and autonomous policy learning under uncertainty	High training complexity and sensitivity to reward design	Medium	Dynamic terrain, sparse reward signals, real-time constraints	Autonomous exploration, multi-step planning, resource allocation

From a deployment perspective, supervised learning is frequently the most developed choice for perception-focused tasks; nonetheless, it is susceptible to distributional alterations prevalent in disaster scenarios (e.g., smoke, debris, thermal anomalies, or unconventional perspectives). Learning from Demonstration (LfD) offers a pragmatic approach for encoding expert tactics in navigation and manipulation; yet, its efficacy is significantly contingent upon the availability and quality of demonstrations, as well as the transferability across various platforms and situations. Reinforcement learning can produce resilient adaptive strategies for exploration and planning; nevertheless, it generally necessitates comprehensive simulation pretraining, meticulous reward shaping, and clear safety limitations to be effective in time-sensitive search and rescue operations. In practice, hybrid pipelines are often advantageous, such as integrating supervised perception with reinforcement learning-based decision layers or employing demos to initialize rules that are subsequently modified through interaction.

### 3.5 Key challenges in H2M learning

#### 3.5.1 Partial view problem and feature variability

SAR robots often function under significant vision limitations due to debris, smoke, inadequate lighting, and fluctuating surroundings, leading to incomplete perspectives and considerable feature fluctuation. H2M learning models frequently falter in these circumstances, erroneously selecting survivors due to occlusion or heat interference. Recent research tackles these difficulties by amalgamating deep learning with multi-sensor fusion, encompassing RGB, thermal, LiDAR, radar, and inertial sensing (El Ahmar et al., 2023; Nobis et al., 2019; Fan et al., 2025). The authors Esteves Henriques et al. (2024) improved YOLOv8n-Pose by inferring absent body keypoints through symmetry, facilitating occlusion-aware detection, while Kalman filtering and homography-based elevation estimation bolstered UAV-ground robot collaboration; however, dependence on RGB constrained performance in low-light conditions.

Occlusion and variations in posture further necessitate few-shot learning and sensor-specific approaches. This study Xiao et al. (2022) utilized few-shot feature fusion and 2D-3D mapping for partial-view detection, whereas LiDAR-based methods like BtcDet (Xu Q. et al., 2022) deduced concealed structures using learned priors but exhibited susceptibility to dust and signal degradation. Thermal and infrared imaging enhance detection in low-visibility conditions (Tavasoli et al., 2025; Song et al., 2025), but are prone to false positives and exhibit restricted structural awareness. Fusion strategies at the pixel, feature, and decision levels (Bustos et al., 2023), in conjunction with multimodal datasets that integrate optical and thermal imagery (Broyles et al., 2022), alleviate sensor constraints. Although reconstruction-based methods using ground-penetrating radar (Hu et al., 2022), occlusion-aware snake robots (Jadeja et al., 2024), and multimodal Sense-Plan-Act frameworks (Tong et al., 2024) enhance perception, reliable performance under heavy occlusion remains elusive.

##### 3.5.1.1 Critical analysis and deployment implications

A comparison of multimodal perception strategies indicates that no single sensing modality is sufficient for reliable SAR operations under degraded environmental conditions. RGB imagery provides valuable structural information, whereas thermal, LiDAR, radar, and inertial sensing improve perception in darkness, smoke, and partially occluded environments. Nevertheless, each modality introduces its own limitations. Thermal sensors remain susceptible to false detections from non-human heat sources, LiDAR and radar performance can deteriorate in dusty or sparse environments, and multimodal fusion frameworks often impose computational and energy demands that exceed the capabilities of lightweight SAR robots. Consequently, the practical challenge extends beyond improving detection accuracy to establishing when sensor observations are sufficiently reliable to support time-critical rescue decisions. These findings highlight the importance of combining uncertainty-aware perception, efficient multimodal fusion, and collaborative cross-agent sharing of complementary observations to achieve robust and dependable scene understanding in challenging disaster environments.

#### 3.5.2 Data contamination and distortion in learning

SAR robots frequently experience data contamination, insufficient domain-specific training, and learning biases that impair their performance in actual disasters. Models trained on non-SAR datasets may erroneously detect debris or thermal reflections as survivors, resulting in false positives, postponed rescue operations, and diminished dependability in low-light, cluttered, or obscured situations. These constraints underscore the necessity for ongoing validation and adaptive learning beyond mere static dataset augmentation.

Data contamination in SAR often comes from putting the wrong labels on things, having inadequate metadata, and using general-purpose datasets. This research Ro et al. (2024) demonstrated that cloud-based vision systems misclassify disaster-specific features, prompting the development of curated labeling pipelines utilizing GloVe embeddings and TF-IDF scoring. While augmentation-based methodologies enhance diversity, they frequently overlook domain adaptability (Alnabulseih et al., 2025). While adaptive multi-sensor fusion with continuous self-checking increases robustness in real-world deployments (Cai et al., 2025), many existing systems exhibit persistent bias due to their dependence on pre-disaster datasets (e.g., COCO García-Samartín et al. (2024)) and synthetic probability maps in deep reinforcement learning (Ewers et al., 2025a). In multi-agent SAR, curriculum-trained MARL enhances exploration but lacks empirical validation (Cao et al., 2024), whereas simulation-based swarm recovery methods exhibit restricted adaptability (Phadke and Medrano, 2024), highlighting the necessity for domain-specific training, real-world assessment, and adversarial robustness evaluation.

##### 3.5.2.1 Critical analysis and deployment implications

Data contamination in SAR extends beyond incorrect labels and reflects a broader challenge of transferring models from controlled training environments to unpredictable disaster scenarios. Domain mismatch, synthetic bias, and insufficient disaster-specific validation frequently limit the reliability of learned models once deployed in real-world operations. Although techniques such as data augmentation, adaptive sensor fusion, and curriculum-based multi-agent reinforcement learning improve resilience, their effectiveness often depends on training conditions that closely resemble deployment environments. As a result, systems trained on generic datasets or simulation-based scenarios may struggle to cope with occlusion, environmental noise, and rapidly changing terrain encountered during actual rescue missions. Rather than focusing solely on scaling training data or model complexity, future research should prioritize domain-aware datasets, continual adaptation, adversarial robustness evaluation, and collaborative cross-agent verification to improve generalization and operational reliability under uncertain disaster conditions.

#### 3.5.3 Guaranteeing correct learning and error minimization

The reliability of SAR robots is frequently compromised by unbalanced training data, misclassifications, and insufficient error verification, resulting in erroneous detections and diminished human confidence. Consequently, uncertainty estimate and confidence rating are crucial for initiating human validation in instances of low confidence. Although previous research has investigated trust, autonomy, and intent inference in SAR systems (Chitikena et al., 2023; Huang et al., 2023), uncertainty-aware learning is still inadequately researched. Current methodologies mitigate errors via confidence filtering, optimization, or verification (Jadeja et al., 2024; Morin et al., 2023; Kashyap et al., 2023; Hendry et al., 2023), although the majority are deficient in adaptive mechanisms that integrate uncertainty and human feedback.

RL enhances error minimization by facilitating adaptive decision-making in dynamic SAR situations, however limits remain. Hierarchical RL frameworks enhance energy-efficient decision-making (Ramezani and Amiri Atashgah, 2024), whereas the integration of RL with LLMs increases flexibility via human feedback (Panagopoulos et al., 2024). Adversarially robust multi-agent RL systems enhance coordination amongst noise (Rahman et al., 2022), while adaptive RL models integrate uncertainty estimation for evacuation planning (Zhao H. et al., 2024). Nevertheless, numerous RL-based SAR systems continue to exclude confidence-aware survivor identification and human-in-the-loop correction.

##### 3.5.3.1 Critical analysis and deployment implications

Reliable decision-making in SAR depends not only on accurate predictions but also on the system’s ability to quantify uncertainty, adapt to limited feedback, and remain resilient against adversarial disturbances under rapidly changing operational conditions. Although reinforcement learning and optimization-based coordination have demonstrated strong performance in controlled environments, their robustness often deteriorates when faced with limited visibility, sensor noise, delayed reward signals, or ambiguous survivor detections that characterize real disaster scenarios. Confidence filtering and verification mechanisms have improved operational safety; however, most existing approaches still treat uncertainty management and human supervision as secondary components rather than integral elements of the learning process. Consequently, achieving an appropriate balance between autonomous decision-making and dependable human oversight remains a key challenge for safety-critical SAR missions. Advancing beyond this limitation will require the integration of uncertainty-aware policy learning, confidence-guided human intervention, adversarial robustness, and collaborative cross-agent verification into a unified decision-making framework capable of supporting reliable field deployment.

## 4 Machine-to-machine learning in SAR

M2M Learning represents the autonomous mechanism by which machines or robots acquire knowledge from other machines or robots without direct human involvement. This involves the sharing of knowledge, self-enhancement, and retraining in response to new information or environmental modifications. Within the SAR realm, M2M learning facilitates robotic collaboration, adaptation to fluctuating situations, and real-time decision making through continuous exchange of information, mutual learning from experiences, and improved performance during operations.

### 4.1 Knowledge acquisition through large language models

Efficient M2M knowledge transfer involves the extraction of essential information and its organization into structured representations, which is a limitation of conventional computer vision systems that depend on static object categories and task-specific labels. Recent multimodal large language models (MLLMs) address this limitation by deriving substantial semantic information from visual inputs and encoding it in comprehensible formats, such as natural language or symbolic representations, thereby allowing for effortless reuse across machines and promoting human examination and validation. This study examines three complimentary techniques to enhance the efficiency, interpretability, and reliability of knowledge learning, representation and communication, addressing the challenges of decreasing ambiguity, interpretation costs, and semantic drift during storage and transmission.

#### 4.1.1 Fine-tuning for structured knowledge learning

Fine-tuning large language and multimodal models enables the transformation of raw visual inputs into structured, interpretable, and transferable knowledge representations, essential for reasoning and generalization in dynamic contexts. In contrast to conventional ontology-driven pipelines, contemporary methods explicitly correlate visual regions with language tokens to acquire relational and compositional semantics. Some researchers (Chen et al., 2024; Peng et al., 2023) illustrate that region-language supervision is crucial for structured visual grounding, transcending global image-text alignment and enabling models to correlate entities, attributes, and relations in a manner conducive to downstream reasoning and reutilization.

Subsequent developments expand organized fine-tuning to encompass more adaptable and interactive environments. You et al. (2023) present enhanced region abstractions to mitigate hallucination, whereas Zhang H. et al. (2024) incorporate conversational supervision for grounded dialogue. Pixel-level and multi-task grounding augment precision and expressiveness using segmentation-aware and unified multimodal learning frameworks (Lai et al., 2024; Rasheed et al., 2024). These research collectively demonstrate that geographically and conversationally anchored fine-tuning is essential for acquiring reliable, interpretable knowledge that can be communicated between machines in time-sensitive SAR situations.

#### 4.1.2 RAG for structured knowledge retrieval

Retrieval-augmented generation (RAG) for structured knowledge retrieval stresses the use of semantic, geographic, and constraint-aware frameworks to organize retrieved information so that valid reasoning can happen in complicated SAR settings. Ontology-guided RAG frameworks ensure domain consistency by organizing retrieved context using clear symbolic relations. This allows for fact-based reasoning and rule-compliant decision support in situations when safety is very important (Sharma et al., 2025). Complementary approaches combine RAG with carefully adjusted language models and feedback from people to help robots make decisions that are flexible and based on knowledge. This includes correction mechanisms based on regret that make long-term collaboration better (Fazlollahtabar, 2025).

Structured retrieval is extended to long-horizon and geographically grounded problems by recent embodied RAG systems. Progressive RAG supports adaptive planning by allowing robots to gradually learn task and scene-specific information without ground-truth demonstrations Xu W. et al. (2024). Semantic-spatial and hierarchical memory architectures arrange multimodal observations into structured representations that support natural language interaction, navigation, and reasoning in a variety of contexts (Xie et al., 2024; Mao et al., 2025). Feedback-aware task decomposition under uncertainty is made possible by integrated embodied LLM frameworks, which further ground retrieved knowledge into executable action primitives (Mon-Williams et al., 2025).

#### 4.1.3 Few-shot prompting/prompt engineering for rapid knowledge acquisition

Few-shot prompting facilitates swift adaptation in SAR robotics by permitting models to integrate mission-specific context via a limited number of structured examples, without necessitating retraining. Safety-conscious prompting systems incorporate verification layers that assess LLM-generated actions against domain restrictions, therefore diminishing hallucinated or hazardous behaviors (Althobaiti et al., 2024). Edge-deployed systems illustrate that prompt engineering can derive real-time contextual insights from multimodal sensor data within stringent resource limitations, providing a streamlined alternative to fine-tuning and establishing a benchmark for more intricate frameworks such as RAG (Gurunathan et al., 2025). Few-shot prompting facilitates structured reasoning, exemplified as chain-of-thought prompting, which allows big models to execute multi-step inference via clearly delineated reasoning processes (Wei et al., 2022).

Prompt engineering has been utilized in robotic perception, planning, and control by directing models to engage in sequential information-seeking and decision-tree reasoning inside intricate contexts (Jiang et al., 2024). The design of natural language tasks further empowers LLMs to function as efficient decision-makers for public-safety UAVs, facilitating swift task reconfiguration at the network edge (Aledhari et al., 2020). Recent assessments demonstrate that the efficacy of prompt-based planning is closely linked to dataset alignment, and that prevalent prompting strategies do not reliably enhance embodied reasoning (Akiyama et al., 2024). Strategies for structured prompt selection and augmentation can enhance efficiency and reasoning quality, however they remain susceptible to the design and ordering of examples (Wu O. T. et al., 2024).

#### 4.1.4 Critical analysis and deployment implications

Current approaches to structured knowledge acquisition in SAR demonstrate that no single strategy adequately balances semantic accuracy, adaptability, and computational efficiency. MLLM fine-tuning, retrieval-augmented generation (RAG), and few-shot prompting each contribute distinct advantages by improving semantic grounding, contextual memory, and rapid mission-specific adaptation. However, their effectiveness depends heavily on stable visual-language alignment, reliable retrieval sources, and well-designed prompting strategies. These assumptions are often challenged in disaster environments characterized by incomplete observations, noisy perception, intermittent communication, and evolving mission objectives. Fine-tuning generally produces the most robust domain-specific representations but requires substantial annotation effort and computational resources. In contrast, RAG facilitates knowledge reuse but remains vulnerable to retrieval inconsistency and knowledge drift, while prompting offers lightweight adaptability at the cost of increased sensitivity to prompt design and hallucination. Rather than relying on any individual technique, future collaborative SAR systems should integrate uncertainty-aware grounding, synchronized cross-agent memory, retrieval verification, and safety-aware prompting to achieve more reliable and resilient knowledge sharing in dynamic disaster environments.

#### 4.1.5 Deployment and safety considerations for LLM-Enabled SAR systems

Large language models (LLMs) are increasingly explored for high-level reasoning, knowledge extraction, and coordination support in collaborative search and rescue (SAR) robotics. However, deploying LLM-enabled components in safety-critical disaster environments introduces practical risks related to reliability, grounding, and real-time constraints. Unlike conventional perception modules, LLMs may generate plausible yet incorrect outputs, which can be harmful if used directly for mission-critical decisions. Therefore, safe deployment requires additional validation layers including sensor grounding, uncertainty checks, human oversight, and auditable logging.

To better illustrate the safeguards required for safe deployment of LLM-enabled SAR systems, Figure 4 presents a simplified validation pipeline integrating grounding, uncertainty assessment, and human confirmation before action execution.

**FIGURE 4:**
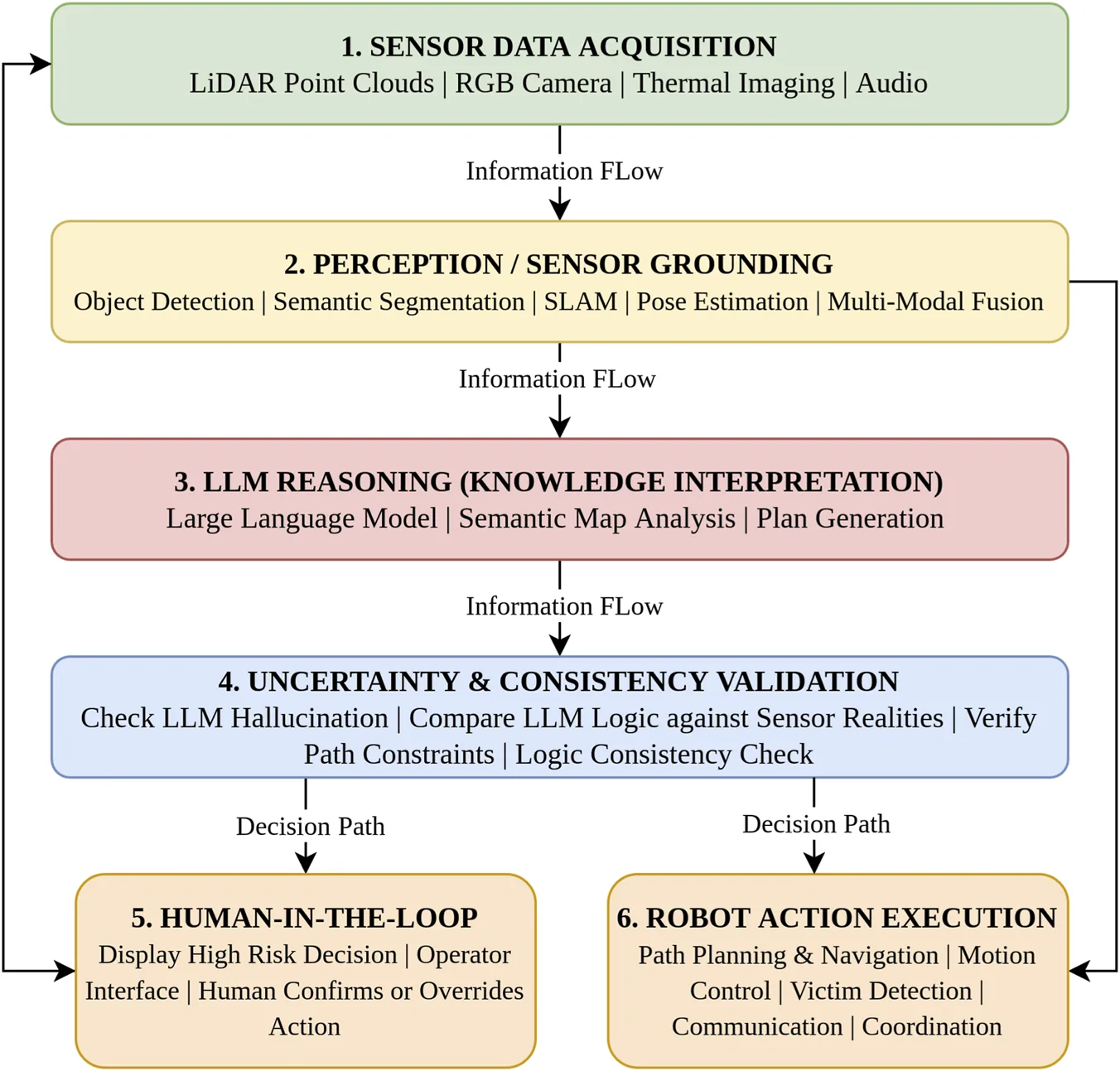
Safety validation pipeline for LLM-enabled SAR systems illustrating sensor grounding, LLM reasoning, uncertainty validation, human confirmation, and action execution stages.

Hallucination Risk in Victim Detection: LLM-based modules may hallucinate objects, events, or semantic interpretations when reasoning from incomplete, noisy, or ambiguous inputs. In SAR contexts, hallucinated victim cues or incorrect hazard descriptions can misdirect rescue teams, cause wasted exploration effort, or trigger unsafe navigation. This risk is amplified when LLMs operate on compressed textual summaries rather than raw sensor evidence. As a result, LLM outputs should be treated as *hypotheses* that must be validated against sensor-derived evidence (e.g., thermal confirmation, visual detection confidence, map consistency) prior to execution.

Need for Sensor Grounding: To reduce ungrounded reasoning, LLM outputs should be constrained by explicit perceptual evidence. Grounding mechanisms can include structured scene graphs, semantic maps, VLM-generated captions tied to specific images, or retrieval from robot-maintained knowledge bases and logs. In practice, grounding means that critical assertions (e.g., “victim detected at location x ”) must be supported by sensor observations and measurable confidence signals. Such grounded representations also improve consistency across robots by enabling shared, machine-interpretable knowledge rather than free-form language alone.

Latency and Deployment Constraints (Cloud vs. Edge): SAR operations often require rapid responses under intermittent connectivity. Cloud-based LLM inference can introduce high and variable latency due to network delays and server-side processing, which may be unacceptable for time-critical decisions (e.g., obstacle avoidance, immediate hazard response). Edge deployment reduces network dependence but is constrained by onboard compute, memory, and power budgets. Practical systems often adopt hybrid strategies: lightweight edge reasoning for immediate actions and cloud-assisted processing for non-urgent planning or report generation. Latency-aware design should explicitly define which decisions are permitted to depend on remote inference and which must remain fully onboard.

Human-in-the-Loop Safeguards: Human oversight is essential when LLM-generated reasoning affects operational decisions. A human-in-the-loop (HITL) policy can be triggered by uncertainty thresholds, conflicting multi-robot reports, or safety-critical action proposals. Operators can approve, reject, or request additional sensing actions (e.g., re-scan with thermal, capture another viewpoint) before execution. This supervision improves trust calibration and reduces the chance that erroneous LLM outputs lead to unsafe behavior, while still allowing LLMs to provide high-level decision support.

Logging and Provenance Tracking: For accountability, debugging, and post-mission analysis, LLM-enabled SAR systems should maintain detailed logs capturing input prompts, retrieved evidence, intermediate reasoning artifacts (when available), uncertainty scores, human approvals, and final actions. Provenance tracking enables teams to trace how specific conclusions were derived and to identify failure modes such as hallucinations, missing evidence, or delayed responses. In safety-critical deployments, such audit trails are also important for operational review, compliance processes, and system improvement across missions.

### 4.2 Knowledge transfer techniques for machine-to-machine learning

In collaborative multi-robot systems, transferable knowledge may include learned policies, feature embeddings, semantic maps, task models, and environmental representations derived from prior mission experiences. Federated learning (FL), knowledge distillation, multi-agent reinforcement learning, and swarm intelligence (bioinspired learning) are among the several learning approaches used in M2M learning. These techniques enable robots to cooperate, adapt, and carry out important duties independently in dynamic and complicated surroundings.

While Section 4.1 examines LLM-based knowledge acquisition in depth given its novelty and the pace of recent development, the following subsections survey four established M2M transfer methods: federated learning, multi-agent reinforcement learning, knowledge distillation, and bio-inspired learning more concisely, with emphasis on their core capabilities and SAR-specific deployment constraints. This allocation reflects the current state of the literature rather than a judgment of relative importance: FL, MARL, KD, and swarm intelligence remain the practical backbone of deployed multi-robot SAR systems, and their limitations define the operational boundaries within which LLM-based approaches must eventually function.

#### 4.2.1 Federated learning (decentralized knowledge sharing)

FL facilitates decentralized machine-to-machine information transmission in SAR by allowing robots to share learnt models while safeguarding raw data, hence maintaining privacy under unstable communication conditions. Peer-to-peer FL architectures boost robustness in the absence of centralized infrastructure (Zhou et al., 2023), whereas distributed frameworks increase scalability, although with additional communication and encryption overhead (Ahmed et al., 2025). Although federated reinforcement learning promotes the exchange of navigation and obstacle-avoidance policies among robots (Na et al., 2023; Nair et al., 2022), aggregation-based updates can suppress responsiveness to local and evolving environments. Extensions pertain to zero-shot motion planning (Yuan et al., 2024), UAV coordination (Nobrega et al., 2024; Kaur et al., 2024), multimodal perception (Pan et al., 2024), and security vulnerabilities (Zia and Haleem, 2025), whereas interpretable decision-tree FL enhances transparency at the expense of flexibility (Heiyanthuduwage et al., 2024).

In SAR deployment terms, the central limitation of FL is not privacy or scalability but aggregation instability under non-IID data distributions, when aerial and ground robots observe fundamentally different environments, globally averaged model updates may benefit neither robot type, making adaptive aggregation strategies a more urgent research priority than architectural extensions.

#### 4.2.2 Multi-agent reinforcement learning (MARL)

MARL facilitates synchronized decision-making in SAR by permitting robots to exchange partial observations and collaboratively optimize policies in dynamic, partially observable settings. Asynchronous and transformer-based multi-agent reinforcement learning frameworks tackle real-world latency and scalability challenges (Yu et al., 2023; Wang et al., 2024), whereas reconfigurable and safety-conscious methodologies enhance adaptability and regulatory adherence (Zhao R. et al., 2024; Gu et al., 2023). MARL improves robustness via behavioral cloning, recurrent architectures, and adversarial training with constrained communication (Lee and Lee, 2021; Sadhu et al., 2020; Rahman et al., 2022). Hierarchical and knowledge-reuse methodologies diminish training expenses and enhance transferability across tasks (Gao et al., 2021; Le et al., 2021), facilitating scalable SAR coordination.

From a deployment perspective, the key constraint on MARL in real SAR operations is not policy expressiveness but communication reliability, most MARL frameworks assume agents can exchange observations frequently enough to maintain joint policy coherence, an assumption that collapses in disaster environments where communication is intermittent, asymmetric, and often lost entirely at the moments when coordination matters most.

#### 4.2.3 Knowledge distillation (KD)

Knowledge distillation allows the effective deployment of SAR models by shifting perceptual and cognitive functions from large models to more lightweight versions (Gou et al., 2021; Hinton et al., 2015). Collaborative and uncertainty-aware KD enhances robustness, collision detection, and incremental learning while reducing forgetting (Gou et al., 2022; Kwon et al., 2021; Yun et al., 2021). KD facilitates multi-robot exploration, collaborative perception, and adaptation driven by feedback through multi-teacher and context-aware distillation (Wang et al., 2025; Li et al., 2023; Zha et al., 2024). Recent TinyLLM and symbolic KD methods make LLM reasoning smaller so it can be used on the edge, which lets SAR work with low latency and protects privacy (Tian et al., 2024; Viswanadh Kandala et al., 2024; Acharya et al., 2024).

For SAR deployment, the most significant practical contribution of KD is enabling capable perception and reasoning on resource-constrained onboard hardware without cloud dependency, but this benefit is only realized when the distillation process preserves performance under the distributional shifts, sensor noise, and partial observability that characterize real disaster environments rather than the clean benchmark datasets on which most distilled models are evaluated.

#### 4.2.4 Bio-inspired learning

Bio-inspired optimization allows swarm intelligence (SI) to enable decentralized coordination, adaptive path planning, and collective perception in SAR. SI-based approaches make navigation more efficient, formations more stable, and control more energy-aware in situations that are always changing due to disasters (Alqudsi and Makaraci, 2024; Ochs et al., 2024; Härdtlein et al., 2024). Brain-inspired dynamics and feature-learning models augment robustness in the face of disruptions (Xu Z. et al., 2024; Li and Yang, 2024), while privacy-aware swarm communication enhances security and coordination (Alqudsi and Makaraci, 2025). For scalable, predictive SAR decision-making, it is becoming more and more important to combine SI with RL and FL.

Despite these strengths, bio-inspired approaches face a deployment ceiling in heterogeneous SAR teams: swarm algorithms designed for homogeneous agents with identical sensing and mobility capabilities do not transfer straightforwardly to mixed fleets of aerial drones, ground rovers, and aquatic robots, and bridging this gap requires either platform-aware coordination protocols or hybrid architectures that combine swarm-level coordination with robot-specific policy layers.

#### 4.2.5 Evaluation metrics and dataset limitations

Collaborative SAR learning frameworks are evaluated using both operational performance metrics and benchmarking environments that assess robustness, coordination, and adaptability under disaster conditions. Existing studies commonly measure victim detection accuracy, navigation efficiency, response time, exploration coverage, communication reliability, and transfer-learning effectiveness to evaluate multi-robot coordination and mission performance. In addition, simulation platforms and benchmark datasets are widely used to validate navigation, perception, and collaborative learning capabilities before deployment in real SAR environments. Tables 3 and 4 summarize representative evaluation metrics, datasets, and simulation environments used across prior collaborative SAR learning studies.

**TABLE 3:** Representative evaluation metrics used in collaborative multi-robot SAR learning frameworks.

Evaluation aspect	Description	Common metrics	Representative studies
Victim detection accuracy	Measures the ability of robotic perception systems to correctly identify survivors and hazards in SAR environments	Precision, recall, F1-score, mAP, false negative rate	Hassan (2025); Queralta et al. (2020)
Response efficiency	Evaluates how rapidly robots complete rescue objectives and identify victims during missions	Time-to-first-detection, mission completion time, victim localization time	Arnold et al. (2018); Queralta et al. (2020)
Situational awareness improvement	Assesses how effectively robots improve shared environmental understanding and hazard awareness	Semantic map accuracy, hazard coverage rate, map update frequency	Ruan et al. (2025); Bravo-Arrabal et al. (2025)
Knowledge transfer effectiveness	Measures how effectively learned policies or representations transfer across robots and tasks	Post-transfer accuracy, convergence rounds, generalization gap, transfer improvement	Wu et al. (2024a), (2022)
Navigation and exploration performance	Evaluates navigation robustness and exploration capability in dynamic SAR environments	Navigation success rate (SR), SPL, path efficiency, collision rate	Xu et al. (2022b); Shen et al. (2025); Bonetto et al. (2023)

**TABLE 4:** Representative datasets, simulators, and benchmark environments used in collaborative SAR learning studies.

Platform/Dataset	Primary use	Characteristics	Representative studies
Gazebo	Multi-robot navigation and coordination	Physics-based robotic simulator with customizable indoor and outdoor SAR environments	Xu et al. (2022b); Hassan (2025)
AI habitat	Object-goal navigation and embodied AI evaluation	Large-scale navigation benchmark supporting realistic indoor scene exploration	Shen et al. (2025); Bonetto et al. (2023)
HM3D/MP3D	Indoor scene understanding and navigation benchmarking	Photorealistic 3D indoor reconstruction datasets used for embodied navigation tasks	Shen et al. (2025)
Custom disaster simulations	SAR-specific mission evaluation	Simulated disaster environments containing debris, hazards, victims, and dynamic obstacles	Hassan (2025); Queralta et al. (2020)
CIFAR-10/CIFAR-100	Transfer-learning and distributed learning evaluation	Image classification benchmarks used to validate collaborative learning and federated transfer methods	Wu et al. (2024a), (2022)

While simulation environments and benchmark datasets facilitate scalable evaluation of collaborative SAR learning frameworks, many existing studies remain heavily dependent on controlled experimental settings that may not accurately represent real disaster-response conditions. Platforms such as Gazebo and AI Habitat support reproducible benchmarking and rapid experimentation; however, they often simplify environmental uncertainty, smoke, dust, occlusion, structural collapse dynamics, sensor degradation, and communication instability commonly encountered during real SAR missions. In addition, many reported results are obtained using homogeneous robotic platforms and stable computing infrastructure, whereas real deployments frequently involve heterogeneous robots with limited onboard computational resources and intermittent connectivity.

These limitations create a substantial sim-to-real gap between experimental performance and operational reliability. Metrics such as navigation success rate, semantic map accuracy, and transfer-learning improvement may demonstrate promising results under controlled conditions, but they may not fully capture the effects of adversarial environments, degraded sensing, communication loss, human cognitive stress, and time-critical decision-making during actual disaster response. Consequently, reported performance values should be interpreted cautiously when considering real-world deployment. These challenges highlight the need for standardized multi-robot SAR datasets, uncertainty-aware evaluation protocols, robust sim-to-real validation strategies, and large-scale field testing to improve the reliability and trustworthiness of collaborative SAR intelligence systems.

#### 4.2.6 Practical constraints in multi-robot collaboration

Although machine-to-machine (M2M) learning enables autonomous knowledge exchange and coordination among robotic agents, real-world deployment in search and rescue (SAR) environments introduces several operational constraints. Disaster zones often exhibit degraded communication infrastructure, heterogeneous sensing platforms, and unpredictable environmental dynamics. These factors significantly influence the efficiency and reliability of collaborative learning mechanisms across multiple robots (Murphy, 2017; Parker, 2007).

Communication Bandwidth Limitations: To stay aware of their surroundings and work together, multi-robot SAR systems often need to share sensor data, environmental maps, and updates to trained models. But in disaster areas, the network infrastructure is often weak or unpredictable, which makes it impossible to send large amounts of data continuously. For instance, airborne drones and ground robots could send out a lot of optical, LiDAR, or thermal sensor data that is too much for the given bandwidth. To overcome this constraint, new studies focus on semantic communication and edge-based processing, wherein robots locally analyze sensor data and communicate only crucial semantic summaries or compressed representations rather than raw data (Yang et al., 2024; Sun et al., 2025).

Non-IID Data in Federated Learning: Federated learning has arisen as a promising paradigm for decentralized knowledge dissemination among robots, eliminating the need for centralized data collection. Federated learning techniques often presume that data distributions among participating agents are independent and identically distributed (IID). This assumption seldom applies in SAR situations. Robots functioning in various locations may face diverse environmental circumstances, sensory modalities, and work objectives. Aerial robots may take photos of extensive structural damage, while ground robots get close-range navigation data. The diverse observations generate non-IID datasets that may impede convergence and induce bias into globally shared models (Kairouz and McMahan, 2021; Yang et al., 2019). Developing resilient aggregation algorithms and adaptive federated optimization techniques continues to be a significant research challenge for multi-robot systems.

Intermittent Connectivity: Dependable network connectivity cannot be assured during disaster response activities owing to infrastructural damage, signal disruption, or environmental impediments. Robots may experience intermittent communication loss with the team while traversing collapsed structures or subterranean regions. Consequently, collaborative learning systems must facilitate asynchronous updates and delay-tolerant communication methods. In these environments, robots can persist in local learning and subsequently synchronize model modifications when communication is reestablished. Decentralized reinforcement learning and distributed coordination methods have demonstrated potential for sustaining collaboration in intermittent connection scenarios (Zhang et al., 2021; Foerster et al., 2016).

Synchronization and Consistency Challenges: A further practical problem in multi-robot collaboration is the maintenance of uniform world models and knowledge representations among agents. When robots autonomously sense their surroundings and communicate with delays, discrepancies may arise in shared maps, object detections, or interpretations of mission status. For instance, two robots seeing the same victim from distinct perspectives may produce contradictory interpretations due to sensor noise or obstructions. Resolving these disparities necessitates consensus-driven learning methodologies, confidence-aware knowledge integration, and decentralized decision-making protocols that allow robots to harmonize conflicting observations and sustain coherent situational awareness (Olfati-Saber et al., 2007; Shorinwa et al., 2024).

Table 5 highlights how practical deployment constraints influence the design of collaborative learning strategies in SAR robotics, emphasizing the need for bandwidth-efficient communication, robust federated optimization, and consensus-based coordination mechanisms.

**TABLE 5:** Practical constraints affecting multi-robot collaboration in search and rescue environments.

Constraint	Primary challenge	Impact on M2M learning	Operational severity	Typical SAR causes	Potential mitigation strategies	Representative methods/Examples
Communication bandwidth limitations	Limited network capacity for exchanging sensor data and model updates	Restricts continuous sharing of perception data, maps, and learned policies	High	Damaged infrastructure, limited wireless coverage, large LiDAR and visual sensor payloads	Semantic communication, edge processing, compressed data transmission	Semantic communication frameworks, edge inference models
Non-IID data in federated learning	Heterogeneous data distributions across robots operating in different environments	Causes biased global models and slower convergence in decentralized training	High	Different sensing modalities, aerial vs. ground robot observations, diverse terrain conditions	Robust aggregation methods, adaptive federated optimization, domain adaptation	Federated averaging (FedAvg), adaptive federated optimization methods
Intermittent connectivity	Temporary communication loss between robots during exploration	Interrupts synchronization of shared knowledge and collaborative learning updates	Medium	Collapsed structures, underground operations, signal interference	Asynchronous learning, delay-tolerant networking, opportunistic synchronization	Asynchronous federated learning, distributed MARL coordination
Synchronization and consistency challenges	Maintaining consistent world models across distributed agents	Leads to conflicting environmental interpretations and delayed situational awareness	Medium	Sensor noise, delayed communication, partial environmental observations	Consensus algorithms, confidence-weighted fusion, distributed decision protocols	Consensus protocols, distributed map fusion algorithms

### 4.3 Reliability and deployment insights for knowledge transfer

To better illustrate how knowledge is extracted, transferred, and adapted among collaborative robotic agents, Figure 5 presents the overall knowledge transfer pipeline for SAR systems.

**FIGURE 5:**
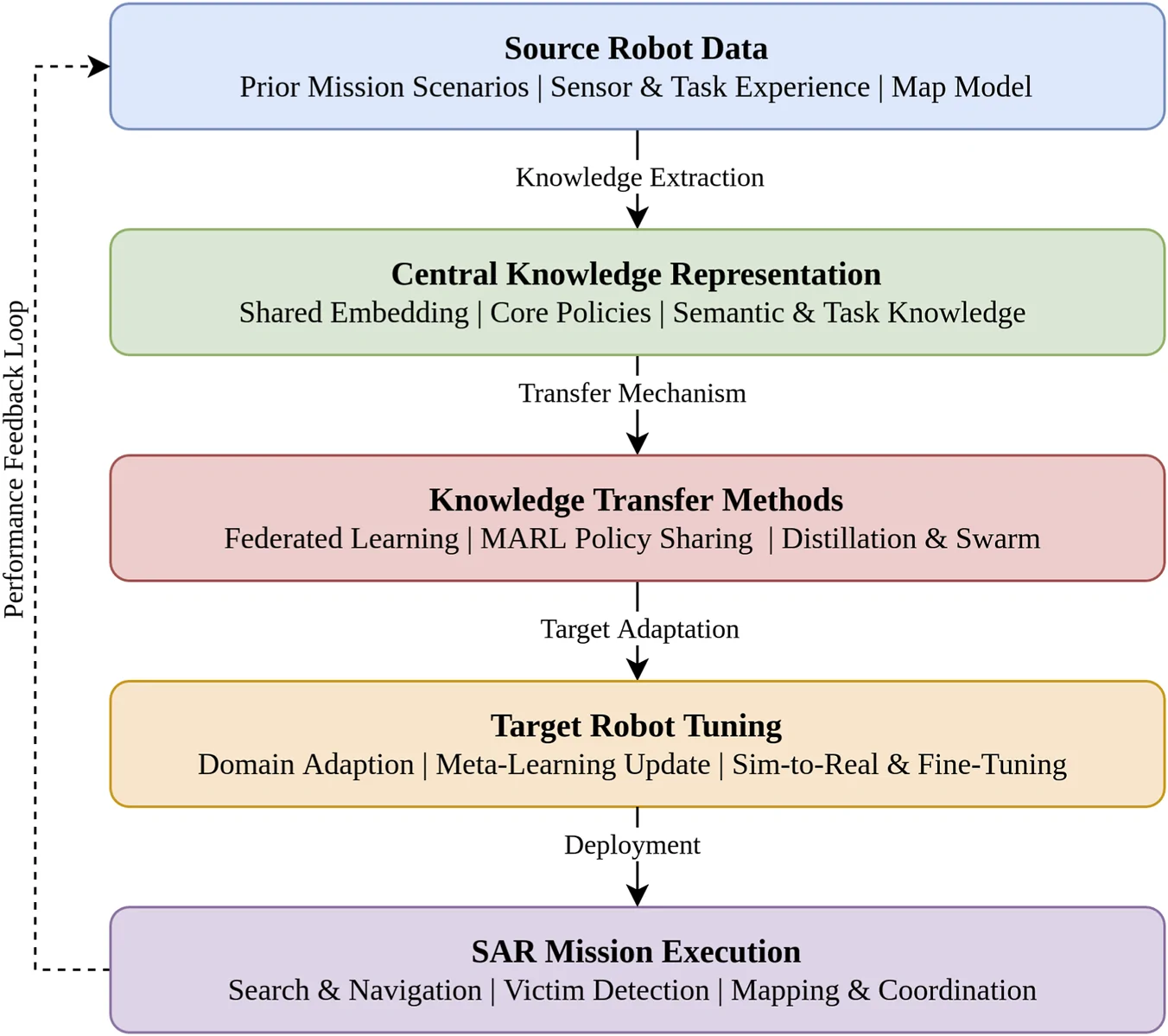
Knowledge transfer pipeline for collaborative SAR robots illustrating knowledge extraction, representation, transfer, adaptation, and mission deployment stages.

#### 4.3.1 Comparative insights: When transfer helps vs. hurts

Knowledge transfer among collaborative robotic agents can significantly improve learning efficiency in SAR missions when robots operate under similar environmental and sensing conditions. In scenarios where multiple robots explore comparable terrains or share overlapping perceptual inputs, transferred representations or policies enable faster adaptation and reduce training overhead. However, transfer effectiveness decreases when the source and target environments differ substantially in terrain structure, sensing modalities, or robot embodiment. For instance, policies learned in structured simulation environments may perform poorly when deployed in highly unstructured disaster environments containing debris, smoke, and unstable surfaces. Consequently, transfer learning in SAR robotics is most effective when environmental dynamics, sensor characteristics, and task objectives remain sufficiently aligned across collaborating robots.

#### 4.3.2 Negative transfer and reliability checks

Although knowledge transfer can accelerate learning across robotic agents, inappropriate transfer may degrade system performance, a phenomenon commonly referred to as *negative transfer*. Negative transfer occurs when knowledge acquired from a source robot or environment fails to generalize to the target robot’s operational conditions. In SAR deployments, this may arise due to heterogeneous robot platforms, differences in sensing modalities, or environmental distribution shifts across disaster sites. Detecting negative transfer requires monitoring performance indicators such as perception accuracy, navigation stability, and task completion success rates during early deployment stages. Practical mitigation strategies include validation of transferred models through simulation rollouts, uncertainty estimation mechanisms, and fallback policies that revert to locally trained models if transferred knowledge degrades system performance.

#### 4.3.3 Sim-to-real and rapid adaptation strategies

Many collaborative learning approaches in robotics rely on simulation environments for initial training because of safety considerations and the high cost of real-world experimentation. However, transferring these models to real-world SAR deployments introduces a significant sim-to-real gap caused by sensor noise, actuator uncertainties, and environmental variability. Techniques such as domain randomization, sensor noise modeling, and incremental fine-tuning with real-world data are commonly used to mitigate this gap. Furthermore, meta-learning and domain adaptation techniques have recently been explored to enable rapid adaptation across heterogeneous disaster environments. Meta-learning frameworks allow robots to learn transferable representations that can adapt quickly to new tasks with minimal additional data, while domain adaptation methods align feature distributions between source and target environments, improving transfer robustness. Table 6 summarizes the common challenges encountered in knowledge transfer for collaborative SAR systems and possible mitigation strategies.

**TABLE 6:** Challenges and mitigation strategies for knowledge transfer in collaborative SAR robotics.

Challenge	Primary cause	Impact on M2M learning	Operational severity	Typical SAR scenario	Potential mitigation strategies	Representative methods/Examples
Negative transfer	Mismatch between source and target environments or robot capabilities	Transferred policies or representations degrade performance instead of improving learning	High	Robots trained in structured environments deployed in debris-filled disaster sites	Transfer validation, uncertainty estimation, fallback policies	Transfer validation frameworks, uncertainty-aware policy transfer
Sim-to-real gap	Differences between simulated training environments and real-world conditions	Learned policies fail under sensor noise, actuation uncertainty, and environmental variability	High	Models trained in simulation encountering dust, smoke, or unstable terrain in real deployments	Domain randomization, sensor noise modeling, incremental real-world fine-tuning	Domain randomization methods, sim-to-real policy adaptation
Heterogeneous robot platforms	Differences in sensing modalities, mobility mechanisms, and onboard computation	Limits transferability of learned representations across robots	Medium	Aerial drones sharing knowledge with ground robots using different sensors	Representation alignment, modular policy architectures, shared latent spaces	Cross-robot representation learning, shared embedding frameworks
Environmental distribution shift	Disaster environments vary significantly across missions and locations	Models trained in one disaster scenario may not generalize to another	Medium	Earthquake vs. flood disaster environments with different terrain and obstacles	Meta-learning, domain adaptation, continual learning	Model-agnostic meta-learning (MAML), domain adaptation frameworks
Communication constraints for transfer	Limited bandwidth and intermittent connectivity during collaborative learning	Restricts frequent exchange of learned representations or model parameters	Medium	Robots operating in collapsed buildings with unstable communication links	Model compression, selective parameter sharing, edge inference	Compressed model updates, edge-based knowledge sharing

#### 4.3.4 Deployment considerations for SAR systems

While knowledge transfer methods improve learning efficiency in collaborative robotic systems, their deployment in real SAR missions must consider operational constraints such as limited communication bandwidth, intermittent connectivity, and heterogeneous robotic platforms. Transferring large models or high-dimensional representations across robots may introduce communication overhead that affects real-time decision-making. Additionally, differences in onboard computational capabilities across robots may restrict the feasibility of complex transfer mechanisms such as centralized training or large-scale neural distillation. Therefore, practical SAR deployments often favor lightweight transfer strategies including selective parameter sharing, incremental updates, and compressed knowledge representations. Incorporating validation mechanisms, uncertainty estimation, and fail-safe fallback policies is essential to ensure reliability and safe operation when transferred knowledge becomes unreliable.

Overall, effective knowledge transfer among collaborative robots can significantly improve mission efficiency in disaster environments by enabling rapid adaptation to new situations and reducing redundant learning efforts. However, practical deployment requires addressing communication limitations, heterogeneous sensing capabilities, and robust validation mechanisms to ensure that transferred knowledge remains reliable in safety-critical scenarios.

### 4.4 Key challenges in M2M learning

#### 4.4.1 Knowledge transfer and sharing between machines

In SAR missions, robots generally function under conditions of partial observability, rendering knowledge sharing crucial for developing a cohesive situational awareness. The exchange of item detections, maps, and acquired strategies enhances coordination, minimizes redundancy, and expedites reaction. Nonetheless, discrepancies in representation or semantics can hinder collaboration, as seen when divergent map interpretations result in erroneous path evaluations. Multiple frameworks facilitate structured M2M knowledge transmission. KT-BT (Venkata et al., 2023) facilitates behavior-tree-based query-response knowledge exchange among robots, promoting dynamic collaboration while depending on predetermined templates. Cross-embodiment transfer has been investigated via latent-space alignment for the dissemination of haptic knowledge (Tatiya et al., 2020) and dynamics-aware trajectory transfer among heterogeneous UAVs without altering baseline controllers (Chen et al., 2020). The transfer of lifelong and implicit knowledge across tasks and morphologies is further examined in LIBERO (Liu B. et al., 2023), non-visual property transfer (Tatiya et al., 2023), and terrain cost inference for multi-legged robots (Prágr et al., 2022).

RL offers an additional method for the acquisition of transferable skills among agents and across various domains. Transfer Expert RL mitigates catastrophic interference during skill transfer (Tommasino et al., 2016), whereas lifelong RL frameworks provide autonomous cross-domain knowledge reutilization through learned projection spaces (Ammar et al., 2015). Deep reinforcement learning has facilitated the transmission of decision policies in extensive, dynamic contexts via correlated feature transfer (Wang et al., 2018). Ontology-driven and cloud-based knowledge repositories enhance the structured sharing of semantic knowledge and execution traces among robots and platforms (Chen et al., 2022; Bozcuoğlu et al., 2018). In addition to explicit representations, shared emotional cues and instructional signals have been examined as implicit conduits for transferable information among agents (Miraglia et al., 2024). MechLMM, a collaborative knowledge framework that integrates large multimodal models, shared knowledge storage, and 3D information mapping to enhance data fusion, object tracking, and contextual information retrieval across multi-robot systems (Lin et al., 2024). Table 7 delineates notable knowledge transfer frameworks pertinent to SAR robots.

**TABLE 7:** Comparison of knowledge transfer approaches across reviewed papers in search and rescue context.

Paper	Model	Knowledge–Type transferred	Transfer direction	Real-time capable	SAR suitability	Key strength
Venkata et al. (2023)	Behavior tree (KT-BT)	Procedural policies	Robot-to-robot	Yes	High	Dynamic, decentralized knowledge exchange
Tatiya et al. (2020)	KEMA	Haptic sensory features	Heterogeneous robot transfer	No	Medium	Embodiment-agnostic transferability
Chen et al. (2020)	Add-on learning signal	Trajectory dynamics	Cross-UAV	Partially	Medium	Controller-safe augmentation
Liu et al. (2023a)	LIBERO benchmark	Task policies	Sequential robot learning	No	Medium	Benchmarking robustness
Tatiya et al. (2023)	Encoder-decoder + KEMA	Object properties	Robot-to-robot	No	Medium	Fast model bootstrapping
Prágr et al. (2022)	Gaussian mixture regressors	Terrain cost mapping	Cross-gait (same robot)	Yes	High	Sparse data adaptation
Tommasino et al. (2016)	Transfer expert RL (TERL)	Task skills	Multi-task within-agent	Yes	High	Preserves past skills modularly
Ammar et al. (2015)	Lifelong RL + projections	Policy + latent spaces	Cross-domain	No	Medium	Cross-domain adaptation
Wang et al. (2018)	DQN + correlated transfer	Dispatch policies	Spatio-temporal	Yes	Low	Real-world deployment
Chen et al. (2022)	Ontology + logic	Semantic task knowledge	Robot-to-robot	Partial	Medium	Structured task assignment
Bozcuoglu et al. (2018)	openEASE (cloud ontology)	Plans + logs + semantics	Cross-platform	Partial (cloud)	Medium	Cloud-based public knowledge
Miraglia et al. (2024)	Natural pedagogy theory	Emotional, shared cues	Human-to-robot	Limited	Medium	Enhances human trust
Lin et al. (2024)	MechLMM + shared 3D map	Sensor + object fusion	Multi-robot sharing	Yes	High	Real-time situation awareness

##### 4.4.1.1 Critical analysis and deployment implications

The effectiveness of M2M knowledge transfer ultimately depends on whether heterogeneous robots can interpret and reuse shared knowledge in a consistent manner. Existing approaches based on behavior trees, ontologies, reinforcement learning, and latent-space transfer enhance coordination by enabling robots to exchange semantic information and transferable policies across collaborative tasks. However, their performance degrades when robots differ in embodiment, sensing capabilities, or environmental observations, leading to semantic ambiguity, incomplete knowledge transfer, and continual-learning challenges. These limitations highlight that the primary obstacle is not the exchange of information itself, but maintaining semantic consistency and policy compatibility across heterogeneous robotic platforms. Addressing this challenge will require robust semantic alignment, verified knowledge synchronization, lightweight policy distillation, and lifelong learning mechanisms that preserve previously acquired knowledge while supporting continual adaptation during multi-robot SAR missions.

#### 4.4.2 Detecting & filtering misinformation in robot communication

In multi-robot search and rescue missions, inaccurate detections or compromised agents can disseminate misinformation, misguiding rescue operations and diminishing team efficacy. This necessitates the development of trust-aware and consensus-driven communication methods. Trust management systems assess agent dependability and reduce adversarial or erroneous behavior by controlling information flow and uncertainty propagation (Vojnar et al., 2024). Human-centered moderation and consensus metrics enhance system coherence when LLM-driven agents provide stochastic or ambiguous outputs (Hu et al., 2025). Trust-aware reflective control allows robot swarms to rectify incorrect actions through weighted communication tactics that correspond with human expectations (Guo et al., 2024). Resilient distributed architectures mitigate misinformation resulting from sensor malfunctions or cyberattacks by facilitating collaborative recovery of localization and state estimation without reliance on centralized infrastructure (Bonczek and Bezzo, 2024; Xue et al., 2024).

Redundancy, consensus, and robust estimates enhance disinformation filtering in decentralized SAR systems. Cooperative localization approaches that are resistant to outliers filter out bad measurements while still being scalable and accurate (Yan et al., 2024). Decentralized active search frameworks integrate detection uncertainty and communication availability into reinforcement learning-based coordination, ensuring operational continuity during communication disruptions (Tabib et al., 2024). Natural language-mediated negotiation and dispute resolution enhance secure and socially compliant multi-agent navigation without necessitating the sharing of global objectives (Mahadevan et al., 2025). In addition to typical misinformation resulting from sensor noise, the transition to LLM-driven communication presents a new risk of adversarial prompt injection. In decentralized M2M networks, a hacked agent may disseminate maliciously constructed semantic prompts intended to corrupt the reasoning of peer robots; for instance, by introducing minor “jailbreak” tokens that compel a recipient to disregard safety limitations or prioritize fictitious threats. In contrast to conventional signal spoofing, these assaults focus on the cognitive layer of the robotic fleet, capitalizing on the LLM’s vulnerability to ambiguous or context-altered directives. Therefore, future M2M frameworks must integrate ‘semantic firewalls’ that authenticate incoming natural language prompts against stringent operational protocols prior to their processing by the receiving agent’s decision engine. Table 8 shows some common ways to find and filter out false information in multi-robot SAR communication.

**TABLE 8:** Comparison of misinformation detection & filtering strategies in robot communication across reviewed SAR papers.

Paper	Framework	Main challenge addressed	Learning type	Trust mechanism	Consensus strategy	Real-time	Key innovation
Hu et al. (2025)	LLM moderation + self-consistency	Misinformation propagation across agents	Multi-agent dialogue learning	Uncertainty scoring	Semantic-based consensus	Partial	Runtime hallucination and knowledge drift control
Mahadevan et al. (2025)	GAMECHAT LLM dialogue	Priority conflict resolution in navigation	Natural language-based negotiation learning	Dialogue-based role validation	Subgame perfect equilibrium	Yes	Role assignment via dialogue for deadlock resolution
Yan et al. (2024)	Robust decentralized cooperative localization	Outlier suppression in sensor measurements	Cooperative localization learning	Residual-based reliability check	No consensus (local filtering)	Yes	Mahalanobis-distance-driven measurement rejection
Xue et al. (2024)	Distributed consensus learning (DCL)	Process noise uncertainty adaptation	Decentralized adaptive learning	Confidence-based trust adjustment	Distributed consensus updates	Yes	Adaptive modeling of localization uncertainty
Bonczek and Bezzo (2024)	Cooperative recovery swarm framework	Localization loss detection and recovery	Behavior-triggered swarm reorganization	Statistical anomaly-triggered trust signaling	Peer-based cooperative recovery	Yes	Mobile landmarking for re-localization without human aid
Vojnar et al. (2024)	Trust management framework (TMF)	Faulty peer identification and mitigation	Trust-based multi-robot learning	Direct and indirect trust updating	Trust-weighted decision fusion	No	Context-sensitive and history-aware robot trust modeling
Tabib et al. (2024)	Confidence-aware active search	Detection uncertainty and false positives	Active exploration learning	Confidence scoring of detections	Implicit confirmation via re-exploration	Yes	Revalidation of low-confidence detections during SAR missions
Guo et al. (2024)	Trust-aware reflective control (Trust-R)	Swarm fault resilience and misinformation isolation	Reflective control learning	Human-inferred trust modulation	Trust-weighted motion consensus	Partial	Dynamic adaptation to compromised or failing swarm members

##### 4.4.2.1 Critical analysis and deployment implications

Maintaining trustworthy shared situational awareness remains one of the most significant challenges in decentralized SAR communication. Existing approaches—including trust-management frameworks, decentralized consensus learning, uncertainty-aware search, and robust localization—improve resilience by helping robots detect and mitigate erroneous observations, adversarial behavior, and sensor noise before information propagates throughout the team. However, these mechanisms become considerably less reliable when operating under partial observability, asynchronous sensing, intermittent communication, or semantically ambiguous information generated by LLM-based interactions. As collaborative systems increasingly rely on language-based communication, additional risks such as adversarial prompt injection, semantic inconsistency, and erroneous reasoning must also be considered. These observations suggest that future multi-robot SAR systems should treat trust verification as a continuous process by integrating trust-weighted communication, semantic consistency verification, uncertainty-aware revalidation, and secure cross-agent reasoning to preserve reliable shared knowledge during dynamic disaster operations.

#### 4.4.3 Learning in unstructured, dynamic environments

In disaster situations, robots work in surroundings that are constantly changing and not well-defined, where hazards, geography, and accessibility might alter at any time. This is why real-time machine-to-machine (M2M) learning is so important. Static or out-of-date shared knowledge may rapidly become dangerous, which shows how important it is to keep learning and sharing information in a way that works for everyone. Recent research focuses on collaborative navigation and interaction in these environments using learning-based coordination. HRMS-Navi (Wang T. et al., 2023) uses hierarchical graph neural networks and attention-based communication to make it easier for multiple robots to navigate in changing settings. In the same way, HGAT-DRL (Zhou et al., 2022) uses different types of graph attention to model complicated interactions between people and robots for crowd navigation, and fuzzy-logic-based controllers let robots move and avoid collisions in spaces that are not well-defined (Abdelwahab et al., 2021). These methods show how localized communication and structured interaction modeling help with coordination when not everything is visible.

Path planning and continuous adaptation enhance resilience in dynamic Synthetic Aperture Radar environments. Hybrid terrain representations that amalgamate grid maps and elevation data enhance the safety of ground robot navigation (Liu J. et al., 2023), whereas planners based on RL incorporate heuristic search, simulated annealing, and recurrent memory to manage unknown or partially observable environments (Pei et al., 2021; Xie et al., 2021; Orozco-Rosas et al., 2022). In addition to navigation, continuous learning frameworks enable robots to adjust in real-time while retaining previously acquired knowledge. Self-supervised traversability estimation with uncertainty awareness enhances stability in dynamic terrain (Yoon et al., 2024), while online active continual learning facilitates lifelong object recognition as classes and domains progress (Nie et al., 2023). Comprehensive research highlights the necessity of tackling catastrophic forgetting and efficiency limitations to guarantee sustained adaptation in autonomous systems (Shaheen et al., 2022). Table 9 delineates a comparison of notable learning methodologies for unstructured and dynamic SAR situations.

**TABLE 9:** Comparison of learning approaches in unstructured and dynamic environments across reviewed SAR papers.

Paper	Framework	Learning type	Transfer medium	Real-time capable	SAR suitability	Key strength
Wang et al. (2023b)	HRMS-navi + GNN	Policy sharing via attention	Robot-to-robot (local GNN)	Yes	High	Efficient decentralized navigation coordination
Zhou et al. (2022)	HGAT-DRL	Behavior via velocity obstacle encoding	Robot-to-robot (graph abstraction)	Partial	High	Incorporates human-aware crowd modeling
Pei et al. (2021)	Improved Dyna-Q	Experience replay with heuristics	Single-agent simulated bootstrapping	Yes	Medium	Fast convergence with adaptive search
Xie et al. (2021)	DRL + RNN (POMDP)	Policy from partial observation	Temporal inference (memory sharing)	Yes	High	Robust to observation loss and latency
Yoon et al. (2024)	ARTE + ERU	Traversability via SSL + replay	Self-replay + local adaptation	Yes	High	Online self-supervised adaptation with uncertainty control
Nie et al. (2023)	OACL framework	Object classification + semantic cues	Selective shared query sampling	Partial	Medium	Label-efficient adaptation across changing classes
Shaheen et al. (2022)	Survey on CL frameworks	Multi-task continual learning	Episodic memory structures	Variable	High	Highlights CL mechanisms suitable for embedded systems
Liu et al. (2023b)	Hybrid terrain map + T-hybrid A*	Terrain-informed path learning	Shared elevation + traversability map	Yes	High	Merges spatial awareness into multi-agent path planning
Abdelwahab et al. (2021)	Dual fuzzy inference	Rule-based motion control	No transfer (standalone)	Yes	Medium	Noise-resilient control in uncertain terrain
Orozco et al. (2022)	QAPF (Q-learning + APF)	Reinforcement-based policy generation	Agent-internal potential-based adaptation	Yes	Medium	Faster convergence and efficient path learning in novel environments

##### 4.4.3.1 Critical analysis and deployment implications

Achieving reliable autonomy in dynamic SAR environments requires robots to adapt continuously without sacrificing the stability of previously acquired knowledge. Existing approaches based on graph-based coordination, reinforcement learning, and self-supervised adaptation improve navigation, exploration, and decision-making by enabling robots to respond to partially observable and evolving environments. Their effectiveness, however, declines when confronted with severe sensor noise, sparse observations, rapidly changing terrain, and continual adaptation demands that increase the likelihood of catastrophic forgetting. These limitations demonstrate that robust field deployment depends not only on adaptive learning but also on preserving long-term knowledge while responding to new environmental conditions. Future collaborative SAR systems should therefore integrate uncertainty-aware experience sharing, continual learning safeguards, and adaptive multi-agent coordination to sustain reliable performance across prolonged and unpredictable disaster missions.

## 5 Machine-to-human learning (M2H) in SAR

In collaborative SAR missions, M2H learning acts as the interpretability interface between autonomous robotic systems and human operators. While H2M learning enables robots to acquire knowledge from human supervision and M2M learning supports knowledge exchange among robots, M2H mechanisms translate robot reasoning, perception outputs, and environmental understanding into human-interpretable information. Effective M2H communication therefore requires balancing interpretability, timeliness, and operational relevance so that explanations enhance situational awareness without delaying mission-critical decisions.

### 5.1 Human-readable knowledge translation

Machine-learned representations, including activation weights or vector maps, are often inscrutable to humans, presenting problems in time-sensitive SAR operations where unarticulated dangers may jeopardize operators. To resolve this, robots must convert internal representations into human-readable formats, including text, annotated photos, or scene summaries, utilizing semantic scene comprehension and captioning. Previous research illustrates this via real-time textual scene descriptions utilizing LLMs and VLMs on UAVs (De Curtò et al., 2023), embodied agents that integrate exploration with captioning for enhanced interpretability (Bigazzi et al., 2023), and semantic-prompt-driven navigation frameworks (Sathyamoorthy et al., 2024). Alternative methodologies amalgamate language with localization or perspective selection to augment situational awareness (Ge et al., 2022; Tan et al., 2022). Visual annotation methodologies, such as segmentation, heatmaps, and linguistically elaborate captioning, enhance explainability and navigation (Deepak et al., 2023; Rohitharun et al., 2022; Humblot-Renaux et al., 2022), whereas comprehensive scene-level reasoning has demonstrated superior efficacy in facilitating proactive human–robot collaboration compared to object-centric outputs alone (Fan et al., 2022).

#### 5.1.1 Critical analysis and deployment implications

The value of machine-to-human communication in SAR is determined not only by how much information a robot can generate, but also by how effectively that information supports rapid human decision-making under stressful conditions. Techniques that translate robotic observations into semantic descriptions, annotated visualizations, and context-aware summaries substantially improve situational awareness by making complex environmental information more accessible to operators. Their effectiveness, however, depends on accurate scene understanding, alignment with mission objectives, and reliable communication. In real disaster scenarios, viewpoint limitations, degraded sensing, unstable connectivity, and domain shifts can reduce the quality and timeliness of these explanations, limiting their operational usefulness. These findings highlight the need for explanation frameworks that balance semantic richness with concise, actionable information, enabling operators to make informed decisions without increasing cognitive workload. Accordingly, future SAR systems should emphasize lightweight multimodal explanation methods that adapt to operator workload, environmental uncertainty, and mission urgency.

### 5.2 Explainable AI (XAI) for decision transparency

In high-stakes multi-agent search and rescue missions, efficient machine-to-human learning depends on XAI to convey robotic reasoning amidst time constraints and reduced visibility (Ghose et al., 2025; Uddin et al., 2026; Ullah et al., 2024). In the absence of explicit explanations, operators may override safety-critical decisions such as rerouting to avoid a gas leak. This can jeopardize mission outcomes and erode trust (Lagap and Ghaffarian, 2024). Contemporary SAR systems utilize feature-attribution techniques (e.g., SHAP (Lundberg and Lee, 2017), lime (Ribeiro et al., 2016) to elucidate sensor impact (Negrilă, 2024), visual instruments such as saliency maps and Grad-CAM (Javaid et al., 2025) to emphasize pertinent targets, and symbolic methodologies (e.g., rules, decision trees, counterfactuals) for logical clarity.

Interactive interfaces, such as entropy-based augmented reality overlays and CE-MRS, enhance shared awareness and facilitate bidirectional reasoning (Schneider et al., 2024). To alleviate cognitive burden, systems implement low-bandwidth, on-demand elucidations and affective signaling (e.g., urgent LEDs) in instances of compromised communication. Nonetheless, evidence indicates that explanations do not consistently enhance decision accuracy or calibrated trust (Rong et al., 2023), necessitating thorough assessment utilizing instruments such as the Explainability Scoresheet (Winikoff et al., 2025). Future SAR XAI must provide adaptive, multimodal explanations by utilizing LLMs for interactive reasoning and customizing outputs according to operator workload and mission phase (Javaid et al., 2025; Ullah et al., 2024). Various explainable AI techniques have been proposed to improve the transparency of robotic decision-making processes. These methods differ in terms of interpretability, computational cost, and suitability for real-time SAR operations. A comparison of commonly used explanation techniques and their operational characteristics in SAR environments is summarized in Table 10.

**TABLE 10:** Comparison of explainable AI techniques for machine-to-human (M2H) decision transparency in SAR robotics.

Method	Explanation mechanism	Explanation type	Computation cost	Real-time feasible	Clarity	SAR suitability
SHAP	Feature attribution using shapley value estimation	Post-hoc model explanation	High	No	Very high	Detailed explanation of sensor contribution but introduces latency in real-time operations
Lime	Local surrogate model approximation	Local decision explanation	Medium	Partial	High	Useful for interpreting individual predictions such as victim detection outputs
Saliency/Grad-CAM	Gradient-based visual attention mapping	Visual explanation	Low	Yes	Medium	Efficient for highlighting image regions responsible for perception decisions
Attention-based models	Transformer attention weights	Intrinsic explanation	Low–Medium	Yes	Medium	Supports interpretable reasoning in multimodal perception models
Rule-based explanations	Symbolic reasoning traces	Logical explanation	Low	Yes	High	Suitable for communicating high-level planning decisions to human operators

In SAR environments, explanations are particularly important for safety-critical decisions such as victim identification, path planning through hazardous areas, or conflict resolution between multiple robot observations. For routine tasks such as standard navigation or environmental scanning, providing detailed explanations may unnecessarily increase operator cognitive load. Consequently, adaptive explanation strategies are required, where explanations are triggered primarily for high-risk or uncertain decisions while routine operations remain autonomous.

However, providing detailed explanations introduces additional computational overhead, which may affect real-time performance during time-sensitive SAR missions. Techniques such as SHAP provide highly interpretable explanations but require significant computational resources, whereas attention-based or saliency-based approaches offer faster explanations with reduced interpretability. Consequently, SAR systems must balance explanation fidelity with latency constraints to ensure that explanations remain useful without delaying critical robotic actions.

Evaluating the effectiveness of M2H explanations requires both technical and human-centered metrics. Common evaluation criteria include operator decision accuracy, response time improvement, trust calibration, cognitive workload, and situational awareness enhancement (Doshi-Velez and Kim, 2017; Hoff and Bashir, 2015). In many human–robot interaction studies, quantitative metrics such as task completion time or error rates are combined with subjective measures including NASA-TLX workload scores and trust perception scales to assess the practical value of explanation mechanisms (Hoff and Bashir, 2015; Chen et al., 2018).

#### 5.2.1 Critical analysis and deployment implications

The practical value of explainable AI (XAI) in SAR depends on delivering the right explanation at the right time rather than maximizing explanatory detail. Feature-attribution and symbolic reasoning techniques provide comprehensive insights into robotic decision-making, whereas saliency-based and attention-driven approaches offer faster explanations that are better suited to real-time operations. Selecting the appropriate explanation strategy therefore depends on the operational context. During time-critical missions, highly detailed explanations may increase computational overhead and interrupt operator decision-making by adding unnecessary cognitive burden. Conversely, explanations that are too brief may fail to convey the uncertainty needed for informed intervention. These observations emphasize that explanation strategies should adapt to both mission context and operator needs. Future SAR systems should therefore provide context-aware, adaptive explanations that prioritize high-risk, uncertain, or safety-critical situations while minimizing unnecessary communication during routine operations.

### 5.3 Proactive AI agent

Proactive AI agents play an important role in SAR missions by identifying potential risks or opportunities before explicit human commands are issued. For example, robots may proactively alert human operators about unstable structures, blocked navigation paths, or possible victim locations based on sensor observations. However, excessive proactive behavior may overwhelm operators with unnecessary alerts, especially in time-critical environments. Therefore, proactive assistance mechanisms must balance autonomy and operator workload by prioritizing high-risk events and filtering low-importance notifications. Simulation-driven approaches facilitate this by predicting mission risks using forward simulations and Monte Carlo techniques, enabling robots to foresee failures and issue early alerts effectively (Al-Hussaini et al., 2021, 2020, 2019). Real-time platforms that combine live video with sensor data from many people make proactive awareness even better, but they need a stable connection (Blum et al., 2014). In addition to alerting, proactive M2H learning positions robots as agents that initiate communication as new insights emerge, underpinned by adaptive interaction models, proactive conversational agent taxonomies, and modular LLM-based systems proficient in planning, memory retention, validation, and intent prediction without necessitating retraining (Xue et al., 2021; Deng et al., 2024; Zhang C. et al., 2024; Buyukgoz et al., 2022).

#### 5.3.1 Critical analysis and deployment implications

In time-critical SAR missions, the effectiveness of proactive AI is determined by how well it supports human attention rather than by the number of alerts it generates. By identifying hazards, anticipating potential failures, and communicating emerging risks before operators explicitly request assistance, proactive agents can enhance mission safety and improve situational awareness. Their contribution, however, depends on presenting timely and relevant information without overwhelming the operator. Excessive low-priority notifications may contribute to alert fatigue, increasing the likelihood that critical warnings are overlooked or delayed during high-stress operations. Likewise, poorly timed interventions can disrupt ongoing decision-making and gradually erode confidence in autonomous support systems. These findings underscore the importance of intelligent notification management, where alerts are prioritized according to operational urgency, mission context, and operator workload. Accordingly, future SAR systems should employ adaptive notification strategies that preserve the benefits of proactive assistance while minimizing unnecessary interruptions and cognitive burden.

### 5.4 Interactive human-robot communication

Interactive communication between robots and human operators enables collaborative decision-making during complex SAR missions. Through natural language interfaces, visual dashboards, or augmented reality displays, robots can communicate situational information, explain planned actions, and request human guidance when uncertainty is high. Effective communication mechanisms improve human trust, situational awareness, and coordination across heterogeneous rescue teams. However, communication interfaces must remain concise and interpretable to avoid information overload during high-pressure disaster response scenarios. Recent research utilizes extensive language and vision-language models to facilitate natural language interaction for planning, perception, and task adaptation (Abbas and Beleznai, 2024; Asuzu et al., 2025), while affect-aware dialogue enhances engagement and perceived reliability (Mishra et al., 2023). Structured taxonomies and learning-based command following help organize these interactions in dialogue management frameworks (Reimann et al., 2024; Lynch et al., 2023). RL makes personalized conversational behavior possible (Su et al., 2022). Communication design also plays a role in how well people communicate with each other. Asymmetric and multimodal interfaces help reduce cognitive mismatch and mistakes, and they also make operators more trusting of each other in collaborative settings (Frijns et al., 2023; Andronas et al., 2021).

#### 5.4.1 Critical analysis and deployment implications

The effectiveness of interactive human–robot communication in SAR depends on enabling operators to make informed decisions under uncertainty rather than simply delivering more information. In disaster response, robotic systems generate outputs that range from sensor-confirmed observations to probabilistic assessments and incomplete situational estimates. Communication interfaces must therefore present these different levels of certainty in a way that allows operators to quickly distinguish validated information from uncertain predictions and unresolved observations. Failing to communicate these distinctions appropriately can result in either misplaced confidence in unreliable outputs or unnecessary hesitation toward valuable recommendations. Although natural-language interfaces and multimodal communication improve accessibility and situational awareness, excessive interaction may increase cognitive workload and delay decision-making during time-critical missions. These findings emphasize that effective communication is fundamentally a problem of confidence calibration rather than communication volume. Future SAR systems should therefore adopt confidence-aware interaction strategies that clearly convey reliability, uncertainty, and operational relevance, enabling operators to respond more effectively in dynamic disaster environments.

To better understand the different mechanisms through which robots communicate knowledge and decisions to human operators, Table 11 compares several key machine-to-human interaction approaches used in SAR systems, including explainable AI, proactive AI agents, human-readable knowledge representations, and interactive human–robot communication frameworks.

**TABLE 11:** Comparison of Machine-to-Human (M2H) interaction approaches in SAR robotic systems.

Approach	Primary function	Communication type	Computation demand	Real-time feasible	Human readability	SAR application
Human-readable knowledge representation	Converts robotic knowledge into symbolic or semantic descriptions	Natural language summaries, semantic graphs, rule-based explanations	Low–Medium	Yes	Very high	Mission status reporting, environment summaries, and knowledge sharing
Explainable AI (XAI)	Provides transparency for model predictions and robot decisions	Feature attribution, saliency maps, attention visualization	Medium–High	Partial	High	Explaining victim detection, navigation decisions, and hazard classification
Proactive AI agents	Anticipates operator information needs and suggests actions	Context-aware alerts and predictive recommendations	Medium	Yes	Medium	Early warning systems, mission planning assistance, and risk alerts
Interactive human–Robot communication	Enables real-time dialogue between robot systems and human operators	Natural language interaction, multimodal interfaces, conversational feedback	Medium	Yes	High	Operator guidance, task clarification, and collaborative decision-making

#### 5.4.2 Section-level critical synthesis

Taken together, the reviewed M2H approaches highlight that the effectiveness of collaborative SAR systems depends as much on meaningful human–robot communication as on autonomous robotic capabilities. Human-readable knowledge translation enhances situational awareness, explainable AI strengthens transparency and trust, proactive agents support early hazard recognition, and interactive communication enables informed decision-making under uncertain and evolving conditions. Despite these advances, the central challenge is not simply generating more information, but determining what information should be communicated, when it should be delivered, and how it can best support operators without increasing cognitive burden. The literature consistently suggests that successful M2H systems are those that adapt communication strategies to mission urgency, operator workload, and the reliability of available information. Consequently, the next-generation of SAR platforms should emphasize human-centered communication architectures that integrate trust calibration, alert prioritization, confidence-aware reporting, and context-sensitive decision support to facilitate effective collaboration in complex disaster environments.

## 6 Discussion, open issues and future directions

The reviewed research represents the state of the art in collaborative learning for multi-agent SAR robotics. The following section discusses key insights, limitations, and open issues across the explored learning paradigms.

### 6.1 Discussion

Based on the reviewed literature, collaborative intelligence in SAR robotics can be broadly categorized into three complementary learning paradigms: human-to-machine (H2M), machine-to-machine (M2M), and machine-to-human (M2H) learning. Figure 6 summarizes a conceptual decision framework illustrating when each paradigm becomes most appropriate depending on mission requirements, data availability, and human oversight needs.

**FIGURE 6:**
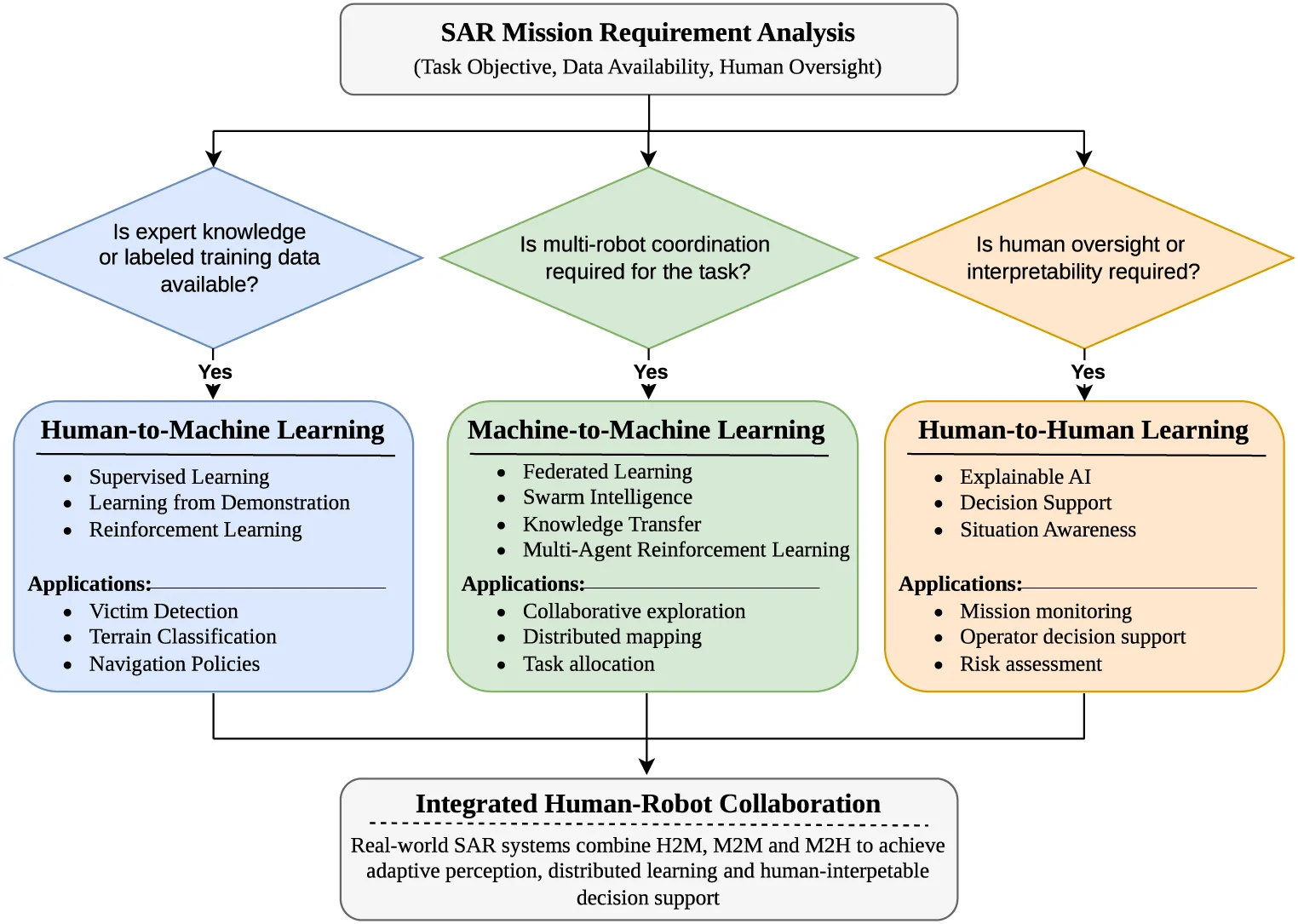
Decision framework illustrating when to apply H2M, M2M, and M2H learning paradigms in SAR robotics.

#### 6.1.1 Human-to-machine learning

H2M learning methodologies enable robots to learn task-specific actions from humans via supervised datasets, imitation, reinforcement learning, or preference-based reward shaping. These solutions allow individuals learn new skills and can make engineering less expensive in dynamic SAR situations. But there are still problems: unclear rewards, little feedback, and low generalizability frequently make it hard to employ in the real world. LLMs have a lot of semantic comprehension, but they do not play a big role in H2M. Structured labeling, demonstrations, and constructed feedback loops are still the main ways that people can transfer knowledge to machines. Prompt-based or language-driven education is not used as much.

#### 6.1.2 Machine-to-machine learning

M2M learning constitutes the foundation of scalable, distributed SAR robotics. Conventional methods depend on federated reinforcement learning, policy distillation, and behavior modularization to disseminate acquired policies and experiences among diverse agents. These strategies enhance team adaptability and diminish dependence on centralized training, yet are constrained by factors such as communication latency, variability in agent capabilities, and non-IID data distributions.

The addition of LLMs to M2M learning adds a new level by allowing for prompt-based semantic knowledge exchange. Instead of sending low-level model parameters or hand-built policy trees, robots can now encode their observations, context, and perception into structured prompts that a common LLM can understand. For instance, robots might work together to classify things using multimodal signals like OCR text, color, and orientation. Then, they might provide semantically enriched object representations to their peers, even if they cannot see the whole thing or it’s blocked. This method helps with both understanding objects and figuring out who owns them, allowing for group reasoning based on symbolic representations rather than just visual features. But current systems still have trouble with grounding prompts, keeping things consistent over time, and making sure that all agents use the same prompt template.

#### 6.1.3 Machine-to-human learning

M2H learning is all about teaching robots how to tell human operators what they are thinking, seeing, and unsure about. This is very important for trust and situational awareness in high-stakes SAR operations. In the early days of M2H, plan visualizations, symbolic rule-based reasoning, or pre-defined discussion templates were widely used. Although these tactics were understandable, they were not very flexible and did not always show the subtleties of new behavior.

The emergence of LLMs enables M2H systems to produce dynamic, context-sensitive natural language explanations based on real-time observations. Robots can employ LLMs to articulate their observations, deduce object characteristics, explain mission progress, or proactively seek validation from human collaborators. This enhances cognitive alignment and diminishes the necessity for predetermined interfaces. Furthermore, LLMs facilitate abstraction and summarization across several modalities, enabling explanations that integrate visual, spatial, and symbolic elements. But we need to be vigilant about the limitations of LLMs. There are safety concerns in mission situations because of the risk of hallucinated outputs, the absence of grounding in sensor data, and the fact that response times are not always predictable. To make sure that M2H interactions are reliable and trustworthy, we need verification frameworks, uncertainty-aware prompting, and human-in-the-loop overrides.

#### 6.1.4 Synthesis across paradigms and emerging challenges

Although H2M establishes the basis for foundational learning, M2M and M2H interactions augmented by LLM-based semantic reasoning enable systems to generalize, collaborate, and elucidate in complex real-world scenarios. The transition to prompt-based reasoning, multimodal correlation, and symbolic representation signifies a novel direction in SAR robots.

Nevertheless, significant constraints persist. M2M frameworks necessitate the standardization of prompt formulation and metadata annotation to guarantee compatibility. LLM-based systems must integrate grounding modules, memory-aware reasoning, and real-time fail-safes to prevent catastrophic miscommunication. In all frameworks, secure ongoing learning, semantic coherence, and agent-level traceability are crucial for transitioning from experimental architectures to functional SAR implementations.

In short, adding LLMs to M2M and M2H systems is a big step forward for collaborative autonomy. It gives robots the ability to think about the world and explain their reasoning to other robots and people. If these improvements are appropriately limited and tested, they could make multi-agent SAR systems much smarter, more open, and more flexible.

It is important to acknowledge, however, that the gap between LLM performance in controlled laboratory or simulation settings and reliable operation in real disaster environments remains substantial and should not be understated. A burning structure with intermittent connectivity, degraded sensors, time pressure measured in minutes rather than hours, and operators managing cognitive overload presents conditions that no current LLM-enabled SAR system has been validated against at operational scale. The studies reviewed here demonstrate promising capabilities in structured benchmarks and constrained experimental settings, but translating these results into trustworthy field deployment requires advances that the field has not yet achieved—including robust hallucination suppression under sensor degradation, guaranteed response latency under communication loss, and validated human-robot trust calibration under acute stress. The contribution of this review is therefore not to suggest that LLM-integrated SAR systems are ready for deployment, but to map the trajectory of a field that is moving rapidly toward that goal while identifying the gaps that must be closed before it arrives.

### 6.2 Open issues

Although there has been progress in robot-to-robot learning and communication at the semantic level, there are still some big problems that need to be overcome. For example, how can robots learn, store, check, and share information in the real world? These unresolved concerns encompass algorithmic design, representational decisions, and system-level safety assumptions.

1. Limits of natural language for symbolic reasoning and inference: LLMs allow prompt-driven symbolic reasoning for concepts like shape, ownership, and spatial relationships. Nonetheless, natural language does not possess the determinism, precision, and formal foundation necessary for safety-critical inference. Ambiguity and underspecification persist as intrinsic dangers, prompting the unresolved inquiry of when language-based thinking may replace logic-based systems and when hybrid symbolic-neural architectures are essential.
2. Natural language vs. structured representations for shared knowledge: Although textual knowledge is adaptable, transferable, and aligned with human understanding, it is less consistent and less queryable than organized databases or knowledge graphs. The efficacy of language-based representations in consistently facilitating conflict resolution, consistency verification, and long-term memory among dispersed robot teams remains ambiguous, particularly in scenarios characterized by partial observability and asynchronous updates.
3. Semantic compression and bandwidth-efficient communication: Language provides an easily understood means for conveying advanced knowledge in contrast to unprocessed sensory information or complete model transfer. Nonetheless, semantic compression presents difficulties in fidelity, ambiguity resolution, and context alignment. An essential unresolved challenge is ascertaining the extent to which task-relevant knowledge may be effectively compressed into symbolic or textual formats without compromising operational reliability.
4. Trust, correctness, and validation of transferred knowledge: Robots do not have strong ways to check incoming knowledge right now, whether it’s model weights, prompts, or symbolic messages. M2M learning could spread old, wrong, or made-up knowledge if it isn’t checked. Confidence-aware validation layers, cross-agent redundancy, and simulation-in-the-loop testing are still not well understood, but they are necessary for safe deployment.
5. Provenance tracking and policy stability under continual learning: As robots learn over time, distinguishing between core policies, peer-acquired knowledge, and self-discovered insights becomes critical. Without provenance tracking and version control, policy regression or unintended overwriting may go undetected. Continual learning systems require layered memory, selective freezing, and explicit attribution to preserve long-term stability.
6. Ambiguity in symbolic grounding under partial observations: Even with shared symbolic descriptions, occlusions and variations in perspective can lead to discrepancies in spatial anchoring (e.g., references like “the crushed bottle near the wall”). Addressing this ambiguity via collective spatial memory, probabilistic grounding, or clarifying discourse continues to pose a significant problem in multi-robot cooperation.
7. Standardization, real-time constraints, and safety of autonomous language use: Current systems do not have consistent schemas for building, processing, and validating semantic prompts across agents, which makes them hard to understand. Also, LLM-based reasoning costs a lot of computing power and isn’t always good for control loops with low latency. Autonomous language production also poses safety hazards, such as hallucination and adversarial prompt injection, which require sandboxed reasoning, backup control mechanisms, and human-in-the-loop protections.
8. Lack of Benchmarks for Semantic Fidelity: Current benchmarks emphasize mission outcomes (e.g., time-to-rescue) rather than the precision of information sharing. The domain lacks standard metrics to quantify ‘semantic drift’—the alteration of meaning during robot-to-robot prompting. Defining precise ‘communication quality’ metrics is crucial for systematically evaluating the dependability of LLM-based coordinating mechanisms.

Despite substantial progress in collaborative SAR intelligence, real-world deployment remains limited by the gap between simulation-based validation and operational disaster environments. Many existing systems are evaluated using idealized sensing assumptions, stable communication, and simplified terrain conditions that may not generalize to smoke-filled, partially collapsed, or dynamically changing environments. Real deployments must additionally address heterogeneous robotic hardware, constrained onboard computation, communication interruption, sensor noise, environmental occlusion, and human cognitive stress during time-critical missions. These operational constraints significantly complicate collaborative learning, coordination reliability, and real-time decision-making compared to controlled simulation settings.

### 6.3 Future directions

Future research must concentrate on scalable, symbolically grounded, and verifiable architectures for knowledge acquisition and transmission, aiming to overcome existing constraints in collaborative learning for multi-agent SAR systems. The following instructions put together the most important things to think about while creating next-generation SAR robots that are strong, easy to understand, and easy to use.

1. Geo-structured symbolic communication for lightweight and privacy-preserving M2M transfer: In resource-limited SAR contexts, robots should utilize organized symbolic representations such as geo-positional prompts to delineate objects, spatial configurations, and their relationships. Observer robots can convey compressed, human-readable scene abstractions without disclosing raw sensor data, while receiver robots reconstruct and juxtapose these symbolic world models with local observations. These representations minimize bandwidth consumption, safeguard privacy, and facilitate symbolic change detection in dynamic environments.
2. Hybrid integration of language with structured knowledge representations: To reduce ambiguity in natural language, forthcoming systems ought to integrate symbolic prompts with structured representations, including spatial-temporal graphs, object attribute mappings, or domain ontologies. This hybrid approach maintains linguistic flexibility while facilitating deterministic querying, grounding, and consistency, which are crucial for coordinating among diverse robots and in partially visible situations.
3. On-device, low-latency reasoning for real-time M2H interaction: Robots have to enable real-time elucidation, inquiry management, and proactive communication through edge-deployable LLMs or symbolic approximations. Bounded-latency reasoning is essential for producing interpretable alarms (e.g., “potential victim behind debris”) and addressing human inquiries during time-sensitive search and rescue phases without dependence on cloud infrastructure.
4. Self-verifying and trust-aware knowledge transfer mechanisms: To stop the spread of wrong or out-of-date information, future M2M learning pipelines should have internal verification modules such semantic agreement scoring, discriminator-based checks, and replay-driven validation. These systems should check how well incoming symbolic knowledge fits with the local context and decide when to accept, improve, or reject information.
5. Memory-aware continual learning with semantic provenance tracking: Robots should always be able to learn new things while keeping important functions through layered memory structures. Semantic tagging of learnt knowledge captures its source, confidence, and version. This enables traceability, rollback, and dispute resolution, helping to prevent catastrophic forgetfulness and unintentional policy drift during long-term deployment.
6. Cross-agent reasoning under partial observability: Future systems have to encourage collaborative thinking on occlusions, geographical gaps, and ambiguous symbolic references. Robots ought to consult peer agents or shared semantic memory and utilize context-aware prompting or geometry-informed reconstruction to deduce item identity, function, or hazard status when local perception is insufficient.
7. Standardized prompt schemas and explainable proactive behavior: Similar to APIs in software systems, defined prompt schemas and symbolic alignment protocols are necessary for reliable inter-agent communication. By producing verifiable causal traces (such as “action taken because expected object was absent”), robots should build on this foundation and demonstrate explainable proactive behavior under uncertainty, allowing for transparent adaptability and human trust throughout developing SAR missions.
8. Development of Semantic Fidelity Benchmarks: Future research must create standards to quantify ‘semantic fidelity,’ assessing whether the receiving robot’s internal model accurately represents the sender’s purpose. Developing open-source datasets of ‘prompt-action’ pairs with accurate interpretations is crucial for assessing the dependability of collaborative semantic systems.
9. Human-aligned knowledge summarization and relay: Robots functioning as semantic observers must condense findings into succinct, queryable reports (e.g., “three heat sources identified, two obscured”) and communicate them to human operators. This connects M2M learning with M2H explanation, enhancing situational awareness and trust in autonomous exploration.

Future research should prioritize large-scale real-world validation of collaborative SAR systems under realistic environmental uncertainty rather than relying primarily on controlled simulation benchmarks. This includes evaluating robustness against sensor degradation, smoke, dust, occlusion, communication loss, dynamic obstacles, limited computational resources, and heterogeneous robotic platforms operating under unstable disaster conditions. In addition, future systems should incorporate uncertainty-aware reasoning, adaptive communication strategies, and human-centered coordination mechanisms capable of supporting decision-making under high cognitive stress and time-critical operational constraints. Developing standardized real-world SAR datasets, sim-to-real transfer benchmarks, and multi-agent field evaluation protocols will be essential for improving deployment reliability, operational trustworthiness, and generalizability across diverse disaster-response scenarios.

## 7 Limitations

Despite providing a comprehensive overview of collaborative intelligence in search and rescue (SAR) robotics, this survey has several limitations. Although a structured search and screening strategy was employed, this work is not intended as a fully PRISMA-compliant systematic review or quantitative meta-analysis. Instead, the final study selection emphasizes representative and influential contributions across H2M, M2M, and M2H learning paradigms rather than exhaustive coverage of all available literature. Quantitative meta-analysis was not performed due to the heterogeneity of experimental setups, evaluation metrics, and simulation environments used across the reviewed studies.

Another important limitation is that many reviewed approaches are primarily validated in simulation or custom experimental environments, which may not fully represent the complexity of real-world disaster scenarios. Reported performance values may therefore not transfer directly to operational SAR settings involving smoke, dust, occlusion, sensor degradation, communication loss, heterogeneous robotic platforms, limited onboard computation, and human cognitive stress. The rapidly evolving nature of large language models and multimodal AI systems also means that some emerging techniques may not yet have mature real-world validation or may not yet be comprehensively represented in the literature. Finally, this survey focuses specifically on collaborative learning paradigms in SAR robotics (H2M, M2M, and M2H) and does not cover other aspects such as hardware design or low-level control systems. These limitations highlight the need for standardized datasets, benchmarking protocols, and more real-world deployment studies in future SAR robotics research.

## 8 Conclusion

This paper examined learning-enabled collaborative frameworks for multi-agent SAR robotics, focusing on human-to-machine (H2M), machine-to-machine (M2M), and machine-to-human (M2H) learning paradigms. Recent advances in reinforcement learning, federated and decentralized learning, semantic communication, and explainable AI show a growing shift toward adaptive, distributed, and semantically grounded SAR systems that can improve coordination, resilience, and situational awareness.

As a structured narrative review supported by a systematic literature search, this work synthesizes representative and influential studies rather than providing a fully PRISMA-compliant systematic review or quantitative meta-analysis. The review highlights key deployment trade-offs across H2M, M2M, and M2H learning, including uncertainty management, communication reliability, trust calibration, explainability, cognitive workload, and continual adaptation.

LLMs and VLMs are increasingly important for symbolic knowledge acquisition, semantic summarization, prompt-based reasoning, and interpretable human-robot communication. However, challenges remain in symbolic grounding, misinformation control, memory stability, proactive communication, and safe knowledge transfer.

Although many reviewed systems report promising results in simulation or controlled benchmark environments, these results should be interpreted cautiously because real SAR deployments involve sensor noise, smoke, dust, occlusion, communication loss, heterogeneous robots, limited onboard computation, and human stress. Future progress requires standardized SAR benchmarks, uncertainty-aware evaluation, robust sim-to-real validation, adaptive communication mechanisms, and large-scale field testing to support trustworthy collaborative SAR intelligence in real-world disaster response.

## Data Availability

The original contributions presented in the study are included in the article/supplementary material, further inquiries can be directed to the corresponding author.
